# The interictal transcriptomic map of migraine without aura

**DOI:** 10.1186/s10194-025-02033-z

**Published:** 2025-05-12

**Authors:** Peter Petschner, Sahel Kumar, Duc A. Nguyen, Dora Torok, Zsofia Gal, Daniel Baksa, Kinga Gecse, Gyongyi Kokonyei, Hiroshi Mamitsuka, Gabriella Juhasz

**Affiliations:** 1 Bioinformatics Center, Institute of Chemical Research, Kyoto University, Uji, Kyoto, 611 - 0011 Japan; 2 Department of Pharmacodynamics, Faculty of Pharmaceutical Sciences, Semmelweis University, Budapest, 1089 Hungary; 3 Center of Pharmacology and Drug Research & Development, Semmelweis University, Budapest, 1089 Hungary; 4 NAP3.0-SE Neuropsychopharmacology Research Group, Hungarian Brain Research Program, Semmelweis University, Budapest, 1089 Hungary; 5 Research Unit for Realization of Sustainable Society, Kyoto University, Uji, Kyoto, 611 - 0111 Japan; 6 Hanoi University of Science and Technology, Hanoi, 100000 Vietnam; 7 Department of Personality and Clinical Psychology, Institute of Psychology, Pazmany Peter Catholic University, Budapest, 1088 Hungary; 8 Institute of Psychology, ELTE Eötvös Loránd University, Budapest, 1064 Hungary

**Keywords:** Migraine without aura, Transcriptomics, Genomics, Drug discovery Molecular pathophysiology

## Abstract

**Background:**

The present study aimed to deliver a replicable transcriptomic map of migraine without aura (MO) and its comprehensive, genome- and drug discovery focused analysis to identify hypotheses for future research- and clinical attempts.

**Methods:**

We recruited 30 controls and 22 MO patients without serious chronic comorbidities/regular medication intake. RNA-sequencing was conducted interictally at two different time points to identify replicable differential gene expression and enriched pathways. Subsequent refining and functional analyses were performed, including: 1) testing additional patient factors, 2) running genetic association analysis on migraine in the UK Biobank (UKB) and our cohort, and 3) predicting drug binding with AutoDock Vina and machine learning to proteins of transcriptomic changes.

**Results:**

Difference in CYP26B1 was identified as key alteration in migraine. Gene set enrichment analysis identified 88 replicated, significant, exclusively downregulated core pathways, including metabolic, cardiovascular, and immune system-related gene sets and 69 leading genes, like CORIN. Logistic regression of leading genes’ and vitamin A pathway-related polymorphisms identified 11 significant polymorphisms in LRP1. Confirmatory analyses excluded a substantial impact of sex, allergy and different vitamin A/retinol intake. Binding simulations and predictions pointed to potential future drug molecules, like tetrandrine and probucol.

**Conclusion:**

The replicable transcriptomic map of MO and functional analyses: 1) identified pathomechanisms related to metabolic, cardiovascular and immune system related processes on a molecular level, 2) reported gene level hits, 3) proposed novel potential etiology, like LRP1-induced decreased retinoic acid signaling, and 4) delivered novel drug candidates for the disorder.

**Supplementary Information:**

The online version contains supplementary material available at 10.1186/s10194-025-02033-z.

## Introduction

Migraine was responsible for about 5% of the total global years lived with disability in 2019 [1] indicating one of the most significant disease burdens. Numerous mechanisms for the disorder and its symptoms have been proposed based on animal models and targeted interventionist approaches [2], yet systematic investigation of the responsible underlying genetic and transcriptomic changes in humans could only provide modest results. Genomic meta-analyses focusing on single nucleotide polymorphisms (SNPs) were able to identify a moderate number of loci, which were in line with both vascular- and neuronal origins of the disease [3, 4]. On a transcriptomic level, human migraine gene-expression studies, investigating mRNAs, were scarcely published [5–10], despite the method being able to capture direction of changes, identify both primary causes and secondary alterations, highlight novel pathophysiology and deliver potential drug targets. These few published mRNA-sequencing (RNA-Seq) and microarray studies could not determine genes that were consistently replicated and/or remained significant after multiple testing correction between migraine patients and controls. Systematic analyses in RNA-Seq studies on a pathway-level yielded few results too. One of them pointed to a downregulation of immune system-related pathways [8]. Another study implicated, for migraine in general, macromolecular complex, nucleus and protein complex-related genes [5], while a non-systematic analysis suggested enrichment of nominally significant genes in mitochondrial processes and inflammatory response between migraine patients and controls [7]. Despite known differences between migraine with and without aura (MO), only one study addressed specifically the more common MO, and found no significant genes after multiple hypothesis correction and only two significant pathways, the macromolecular and ribonucleoprotein complex pathways [5].

A reason for the inconclusiveness could be the underlying heterogeneity of the investigated samples, influencing gene expression, migraine or both. We hypothesised that rigorous collection of a large sample with clinical diagnosis of MO subtype, strict exclusion criteria for serious acute/chronic illnesses and regular medication intake, and correction for remaining phenotypic variables might yield replicable results and provide a reliable transcriptomic map of the disease. Such a map and its functional analysis could widen knowledge regarding the underlying pathophysiology, connection between MO-transcriptomics and -genetics, boost drug development and provide testable hypotheses for clinical interventions and future studies.

**Fig. 1:**
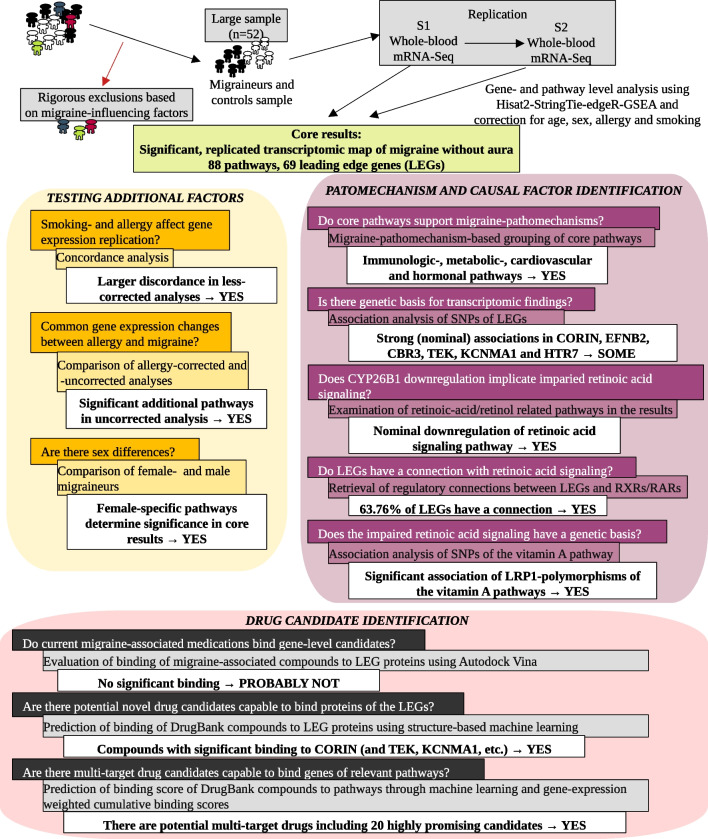
Workflow and Experimental Design for Migraine Transcriptomic Analysis. Figure shows scientific questions, methodological approaches and key findings and implications of the study. Significance, unless otherwise noted, represents significant values after multiple hypothesis correction. Abbreviations: *LEGs* – Leading Edge Genes, *S1* – Time Point 1, *S2* – Time Point 2. *SNP* – Single Nucleotide Polymorphism

To generate such a reliable interictal transcriptomic map of MO, we collected a large, rigorously phenotyped cohort of MO patients and healthy controls, and performed gene- and pathway-level analyses on raw RNA-Seq data of interictal, whole blood samples taken at two independent time points. Various analysis steps and additional datasets were used: 1) to test if correction for remaining phenotypic variables (smoking, history of allergy) influence gene expression replicability, 2) to obtain a differentially expressed core set of genes and pathways for MO, 3) to identify allergy-/sex-dependent genes and pathways in the core set, 4) to determine potential underlying genetic variants behind transcriptomic findings, 5) to characterise the leading gene-level candidate, *CYP26B1*, and its role in the observed changes, 6) to test binding affinity of current medications to the identified genes and 7) search for novel drugs capable of influencing the gene- and pathway-level targets (Fig. 1).

## Methods

### Participants

Volunteer recruitment for the migraine cohort took place through advertisements at university, headache clinics, in newspaper articles and online in Hungary. We collected data about diagnosis of MO, smoking status, age, sex, history of allergy, pregnancy, breast feeding, any other serious acute, previous and chronic illnesses and regular medication intake. Additionally, we collected Migraine Disability Assessment (MIDAS) measures, including attack frequency (MIDAS A), headache severity (MIDAS B) [11], and years since MO onset, from the MO cohort using standardized questionnaires and headache diaries. Diagnosis of episodic MO was made by an expert neurologist based on the International Classification of Headache Disorders 3rd edition (beta version) [12]. Subjects were excluded for 1) neurological problems (except MO), 2) psychiatric disorders, 3) serious, acute, previous and chronic illnesses (except history of allergy), 4) regular medication intake, except contraceptives, 5) pregnancy and breastfeeding determined by self-reported questionnaires and a neurologist. Mental health problems were assessed by Mini International Neuropsychiatric Interview (MINI) [13]. On measurement days, blood was taken from cubital veins of a random subset of participants (*N* = 52, transcriptomic cohort) following the recording of randomly assigned psychological tasks from participants. Sampling and measurements were repeated for a second time with average time difference of around four weeks. Subjects had to confirm that 48 h before sample collection no analgesic, anti-migraine medication and 4 h before no caffeine was consumed. MO samples were excluded if they were not attack- and headache-free 24 h before and after sample collection at both time points. Healthy controls had to be headache free 24 h before sampling.

### RNA sample preparation, sequencing, quality control and gene-level analysis

For purification and extraction of blood samples PAXgene Blood mRNA kit (Qiagen, Venlo, The Netherlands) was used with QiaCube instrument at the Institute of Psychiatry, Psychology & Neuroscience, King’s College London (United Kingdom) per the manufacturers handbook. Samples were processed according to “NEBNext Ultra Directional RNA Library Prep Kit for Illumina” (NEB #E7420S/L) at GenomeScan (Leiden, The Netherlands). Sequencing was performed using Illumina NextSeq 500, with 75 bp single-end sequencing according to Illumina’s and GenomeScan’s standard operating procedures and ISO standards with a sequencing depth of 20 M reads per sample.

Remaining adapters (attached during sequencing) were removed with Trimmomatic (v 0.39) [14]. Raw data quality control (QC) was done by FastQC (v0.11.8, [15]), resulting in a quality score > 30 for all reads, meaning a 99.9% accuracy in base calls. Consequently, no sample exclusions were made.

RNA-seq reads were aligned to Ensembl human reference GRCh38.96 with HISAT2 (v2.2.1, [16]), with default settings and spliced alignment options for downstream transcriptome assembly and rna strandness R, providing alignment rates ranging between ~ 91–96%, except one sample with 84%. Subsequently, StringTie (v2.1.4, [17]) was used for transcriptome assembly and quantification.

Gene level analysis to determine differentially expressed genes was done in R (v4.0.3, [18]) with Bioconductor (v3.11, [19]) package edgeR (v3.30.3, [20]) using the quasi-likelihood F-test. P-values were adjusted for multiple testing by the Benjamini–Hochberg false discovery rate (FDR) [21].

All transcriptomic analyses included covariates: 1) age and sex or 2) age (for sex-specific analyses), where age corresponded to the exact age at the time of measurement (at S1 and S2). In analyses considering the impact of vitamin A intake and using the covariate of supplements with certain or potential vitamin A content we used supplement intake as a binary variable.

For gene set enrichment analyses (GSEA, [22]) we used all C5 gene sets representing Gene Ontology (GO) sets. C5 sets were chosen, because: 1) these provide the largest coverage, 2) represent various functional gene sets able to connect a high-level neurologic disorder with gene expression changes [23], and,3) are the most comparable with other studies on the field. Results using the cellular compartment sets are presented only in supplementary materials, as we considered these hard to interpret with respect to migraine.

### Gene-set enrichment analysis

GSEA was performed with Bioconductor package fgsea (v. 1.14.0, [24]) using default settings with size of gene sets between 15 and 500. Gene sets were obtained from Molecular Signature Database (MSigDB) v.7.4. Results are published for Gene Ontology molecular functions (GOMF) and biological processes (GOBP) sets in the main text.

A pathway was significant if FDR < = 0.05 at both time points. Leading edge genes (LEGs) of the significant GOBP and GOMF pathways, a subset of the pathway’s genes, which appear in the ranked list of genes before the running sum reaches its maximum, were extracted. In short, LEGs were genes driving significant pathway-level enrichment. LEGs were considered important if they have shown a mean absolute logarithm twofold change (Log2 FC) value over 2 (for LEG5) and over 0.5 (for LEG69). Since these genes were important behind the significantly replicated pathways, our criteria were rather as a post-hoc filtering than a standalone statistical evaluation. The thresholds were selected based on literature for 0.5 [25], and conventions in transcriptomic analyses, which usually consider a log2 FC > = 2 a threshold for substantially altered genes.

### Sign concordance analysis

Gene expression values (Supplementary material 2) were filtered for the common set of genes present at all time points and corrections investigated, with the filtered set consisting of 10,765 genes. Log2 FC signs were considered concordant if a given gene had either positive or negative log2 FC at both time points for a given comparison, discordant otherwise. Similar analyses were conducted after filtering for an absolute log2 FC value of 0.001 and 0.05 (Supplementary material 3) to assess genes with increasingly non-near zero expression change.

### UK Biobank participants

Migraine phenotype was determined based on cases, who had migraine as a first medical diagnosis in UK Biobank (UKB) (project ID: 71718). We used field ID: 131052, G43 to determine first reported migraine (resulting in 6,307 migraineurs and 200,571 controls). This phenotype is considered to be a more genetically determined migraine phenotype [26]. Smoking status was defined based on current (field ID: 1239) and past (field ID: 1249) smoking habits. Information related to asthma or hayfever/eczema/allergic rhinitis from medical conditions (field ID: 6152) were used to determine asthma status and estimated food nutrients and vitamin and/or mineral supplement use was utilized for vitamin A- and vitamin A retinol equivalent intake (field IDs: 26061, 20084). We used field IDs 31, 21003 to determine sex and age of participants in UKB. Control individuals were participants who, at the mean age of the migraine group (+ -SD) had no diagnosed disease.

### Migraine cohort

Data from whole migraine cohort including those that were not sampled for transcriptomic analysis (*N* = 289) were included in the genetic analyses. Phenotypic variables were determined similarly for the subset that underwent transcriptomic analysis, resulting in 172 migraine cases and 117 controls.

### Selection of Single Nucleotide Polymorphisms for Leading Edge Genes for genetic analyses

SNPs that belong to the LEGs and their 10 kbp vicinity were retrieved using gene boundaries according to hg19 known gene gene track of UCSC and ANNOVAR [27]. The final list of SNPs and genes can be found in Supplementary material 4.

### Vitamin A pathway genes and selection of Single Nucleotide Polymorphisms for genetic analyses

Vitamin A pathway genes were manually selected from [28] and [29], based on protein names mentioned in the texts, human gene names retrieved using GeneCards [30], and corresponding SNPs extracted like for LEGs (Supplementary material 4).

### Plink2 genetic analyses

Logistic regression of the above SNPs was performed for migraine with Plink2 using covariates, sex, age, top 10 principal components of the genome, smoking, allergy in both UKB and our cohort. Additionally, genotype array was included in the UKB analyses. For vitamin A pathway SNPs, retinol equivalent- and vitamin A intake was also used in the UKB analyses (Supplementary material 1). All genetic analyses used Bonferroni-correction (threshold of significance/number of tests performed) as this is standard on the genomic field [3, 4].

### Binding predictions using AutoDock Vina

Sixty-four migraine medications were selected from DrugBank 5.1 [31] by two pharmacists (SK and KG). Antiemetic and non-small molecule drugs (e.g., antibodies, botulinum toxin, etc.) were excluded (Supplementary material 5). For binding simulations, small molecule 3D structures of the remaining 54 drugs were downloaded from PubChem [32]. For AutoDock Vina analyses pdb files containing AlphaFold2 predicted protein structures were downloaded from UniProt [33]. Proteins and ligands were prepared with default settings using prepare_receptor command (except that “-A” flag was used to add hydrogens to structures) and ml_prepare-ligand command, respectively. Basic docking simulations using 0,0,0 as centre and 126,126,126 as size for grid parameters with Vina forcefield using AutoDock Vina (v1.2.3., [34]) were run with “exhaustiveness” set to 32. Based on preliminary runs maximum number of binding modes was left at default. From the simulated binding pair modes, the one with the lowest score (strongest binding) was selected as representative of the drug-protein binding. Docking simulations require normalization [35]. Normalization was performed by randomly sampling 100 proteins for each drug (Supplementary material 6) and Z-scores for the drugs using the mean and standard deviation of random samples were calculated and one-sided p-values derived from these scores (energetically more favourable binding pairs only). Z-test significance values were considered significant if they survived Bonferroni multiple hypothesis correction (*p*-value < = 1.3617 × 10^–5^).

### Drug-protein binding prediction with machine learning

Genes of the significant pathways and all drugs from the 5.1 version of DrugBank [31] were used. To our knowledge, no immediately deployable comprehensive drug binding prediction pipeline exists for genes, therefore we developed our own (Supplementary material 1). In detail, genes were extracted from all pathways for a relevant comparison, and structures downloaded from the UniProt database [33]. All proteins of a gene were used. HyperAttentionDTI, using a convolutional neural network (CNN) with an attention block and the drug’s SMILES strings and the amino acid sequence code for proteins, was utilized to predict drug-binding scores for all proteins [36]. Key hyperparameter settings were: learning rate of 1 × 10^–4^, weight decay of 1 × 10^–4^, input embeddings of 64. CNN blocks had three stacked 1D-CNN layers with 32, 64, 96 filters, window sizes were 4, 6, 8 for drugs and 4, 6, 12 for proteins, output block was four layers with 1024, 1024, 512 and 2 neurons, respectively. Dropout rate was 0.1 and batch size 32. Predictions were run on a 24 GB Nvidia RTX A5000 GPU.

After obtaining protein-drug prediction for 11,064 drugs from DrugBank, the maximum binding score among all proteins for the gene was associated as the binding score of the drug and the gene. We calculated all drug-gene binding scores and assessed how many times each drug was in the top 10 most binding drugs for every gene from the 9,657 genes. Such drugs are less suitable drug candidates due to their ubiquitous binding, thus drugs with more than 10 times in the top 10 predictions of the 9,657 genes were excluded. Finally, normalisation using the mean and standard deviation of the drug's predicted binding score to all proteins was performed and right-sided p-values were calculated. FDR values were calculated considering binding to all LEGs for a given drug. The values, in sum, show which proteins are bound by the given drug with significantly higher affinity than a hypothetical average protein.

One drug-one target approaches in migraine drugs were criticized for inadequacy to reveal relevant candidates [37]. Thus, a pipeline from the significantly enriched pathways has been developed to obtain pathway level drug candidates. Drug-gene prediction scores for a given gene were multiplied with the mean absolute log2 FC values of a gene from the migraine versus control allergy-corrected comparison, if it had both prediction and gene expression values in all measurements. To calculate effect on a pathway of a given drug, genes belonging to a pathway weighted with the drugs’ normalised binding affinity to an average protein (as in single target case) were aggregated using the L2-norm. The L2 norm gave binding score for each drug on the pathway level and can be considered as a weighted sum of the binding scores with the expression change of the genes in a pathway. Empirical p-values were calculated using the Monte Carlo integral of the ranked p-values list for all pathways and drugs and pathway-gene pairs below the arbitrary significance threshold of 0.0005 are presented.

## Results

### Descriptive statistics

We assessed clinical characteristics and basic demographic data of the transcriptomic cohort summarized in Table 1 (for additional population characteristics such as sleep quality/chronotype, diet, exercise, smoking status, allergy history, supplement intake, and contraceptive use, see Supplementary material 1).

**Table 1:** Demographic and Clinical Characteristics in Control vs. Migraine Groups

Characteristic	Control (*n* = 30)	Migraine (*n* = 22)	Test statistic	*p*-value
Sex (M/F)	14/16	5/17	χ^2^ = 2.1895	0.139
Age (S1) [years]	26.4 ± 4.02 (21–37)	26.8 ± 5.13 (20–37)	U = 324	0.92
Age (S2) [years]	26.6 ± 3.99 (21–37)	26.9 ± 5.17 (20–38)	U = 319	0.847
MIDAS A	not applicable	11.71 ± 5.83 (2–21)	not applicable	not applicable
MIDAS B	not applicable	5.57 ± 1.60 (2–9)	not applicable	not applicable
Duration of MO onset [years]	not applicable	12.42 ± 6.31 (2.55–28.23)	not applicable	not applicable

Data for continuous variables (age, MIDAS, duration of migraine onset) are presented as mean ± SD (range). Sex distribution was compared using a chi-square test between MO and controls, and age was compared using a Mann–Whitney U test at both scan time points (S1, S2). MIDAS (A, B) and duration of MO onset were not tested against controls as these were not recorded in the control group. *Abbreviations: SD* – Standard Deviation, *U* – Mann–Whitney U test statistic, χ^2^ – Chi-squared statistic, *S1* – Time Point 1, *S2* – Time Point 2, *MIDAS* – Migraine Disability Assessment, *MO* – Migraine without Aura

### Smoking- and allergy-correction increase gene expression replicability in migraine

First, we tested if remaining phenotypic factors like history of allergy (henceforth allergy) and smoking status (henceforth smoking), in addition to the standard age and sex variables, could influence replicability of gene expression in general (and if their correction is necessary). Discordant gene expression signs at the two time points (S1 and S2) decreased in the following order of corrections in migraine versus control comparisons: 1) age and sex correction (44.38%), 2) age, sex and allergy (36.01%), 3) age, sex and smoking (21.68%), and 4) age, sex, allergy and smoking (21.39%) using all 10,765 genes. Results were consistent with this pattern if genes with small expression differences between groups were excluded (Supplementary material 2).

### Core gene-level expression changes in migraine implicate CYP26B1 gene

Correction for age, sex, smoking and allergy (henceforth the allergy-corrected analysis) left no significant, replicated genes at both time points (no significant genes at S1 and five at S2) comparing MO and controls (Supplementary material 2). Investigation of log2 FC revealed that *CYP26B1* had the largest average log2 FC in the analyses and was downregulated in MO (log2 FC_S1_ = − 5.865, log2 FC_S2_ = − 5.860, Supplementary material 2).

### Core pathway-level differences in migraine support pathophysiology

GSEA of allergy-corrected expression of the migraine versus control comparison yielded 88 enriched pathways (FDR < = 0.05 at both time points and concordant normalized enrichment score [NES]). All significant, replicated pathways were downregulated (Supplementary material 7). For the list of the top 10 pathways based on their mean NES of S1 and S2, see Table 2.

**Table 2:** Top 10 Significant Gene Ontology Pathways in Migraine Without Aura

Pathway name	Mean NES	FDR at S1	FDR at S2
Myeloid leukocyte mediated immunity	− 2.66	5.44E- 08	4.3E- 08
Myeloid leukocyte activation	− 2.55	5.44E- 08	4.3E- 08
Cell activation involved in immune response	− 2.41	5.44E- 08	4.3E- 08
Defense response to fungus	− 2.20	1.72E- 03	1.1E- 03
Antimicrobial humoral response	− 2.19	4.98E- 04	1.3E- 03
Regulation of DNA templated transcription in response to stress	− 2.19	5.72E- 04	6.4E- 06
Response to fungus	− 2.18	4.20E- 03	2.5E- 04
Regulation of transcription from rna polymerase II promoter in response to hypoxia	− 2.12	3.53E- 03	1.5E- 04
Regulation of cellular amino acid metabolic process	− 2.02	3.25E- 03	7.2E- 03
Activation of innate immune response	− 2.00	1.08E- 03	2.4E- 04

The table shows top 10 replicated significant Gene Ontology pathways for interictal episodic migraine without aura patients compared to controls based on mean normalised enrichment scores using two time points. For detailed statistics, see Supplementary material** 7**. *Abbreviations:* *FDR* – False Discovery Rate, *NES* – Normalized Enrichment Score, *S1* – Time Point 1, *S2* – Time Point 2

**Fig. 2:**
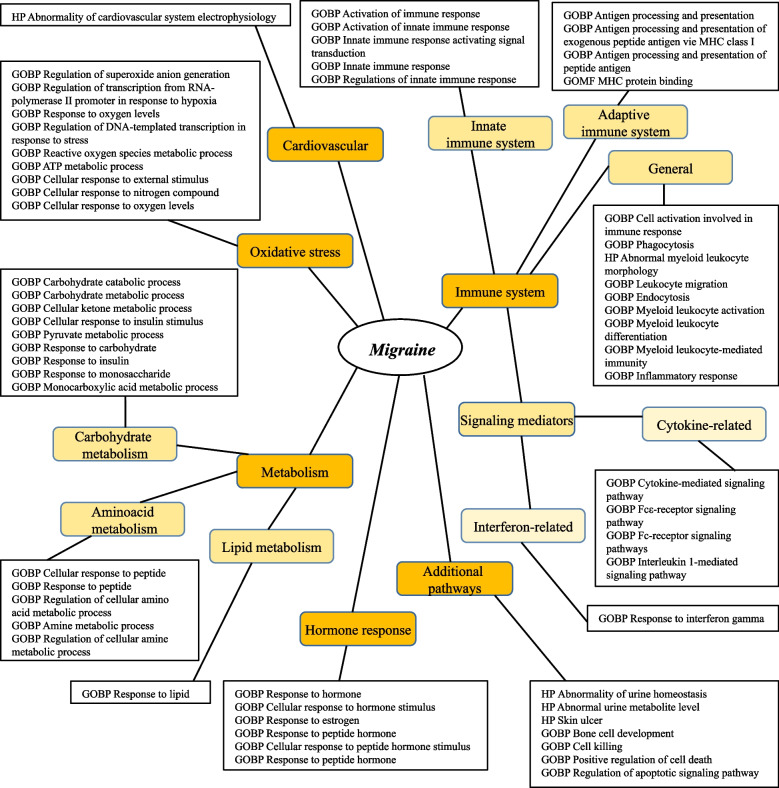
Functional Categorization of Significant Pathways in Migraine Pathophysiology. Functional categories were selected based on expert knowledge. Pathways were assigned to terms based on the relevance of their name/functional description to the given term. *Abbreviations: HP* – Human Phenotype Ontology, *GOBP* – Gene Ontology Biological Process, *GOMF* – Gene Ontology Molecular Function

Grouping of the significant pathways into migraine-related, high-level categories based on the pathway’s functional descriptions is visible on Fig. 2 and support known pathophysiology mechanisms including altered amino-acid-, lipid, carbohydrate metabolism, cardiovascular- and hormonal elements and immune system-related processes.

### Leading edge analysis identifies 69 genes in migraine

To extract genes with relevance for the replicated, significant core pathway-level results, LEGs – genes driving pathway-level enrichment signals – were extracted from pathways. Among the LEGs, five (LEG5) showed a mean log2 FC value below −2, namely: *CYP26B1*, *CORIN*, *PRTN3*, *CCL23*, *CTSG* and 69 (LEG69) showed a mean log2 FC below −0.5. There were no LEGs with positive log2 FC (Table 3, Supplementary material 2).

**Table 3:** Leading Edge Genes in Migraine and Their Expression Changesdant normalized enrichme

Gene name	Mean log2 FC	Log2 FC at S1	*p*-value at S1	FDR at S1	Log2 FC at S2	*p*-value at S2	FDR at S2
*CYP26B1*	− 5.8622	− 5.8646	0.0001	0.2005	− 5.8598	0.0001	0.0585
*CORIN*	− 4.1653	− 3.5429	0.0015	0.5275	− 4.7877	0.0004	0.1426
*PRTN3*	− 3.0534	− 3.3806	0.0005	0.2991	− 2.7263	0.0029	0.4484
*CCL23*	− 2.7878	− 2.7640	0.0386	0.8735	− 2.8115	0.0458	0.8297
*CTSG*	− 2.6687	− 2.7865	0.0004	0.2936	− 2.5509	0.0009	0.2410

Table shows gene level expression statistics of leading edge genes with absolute mean log2 FC value > = 2 (LEG_5_) of allergy-corrected analyses, for detailed values see Supplementary material 2*. Abbreviations: Log2 FC* – Logarithm Two fold Change, *FDR –* False Discovery Rate, *S1 –* Time Point 1, *S2* – Time Point 2

### Influence of allergy and sex on core gene expression results

**Fig. 3:**
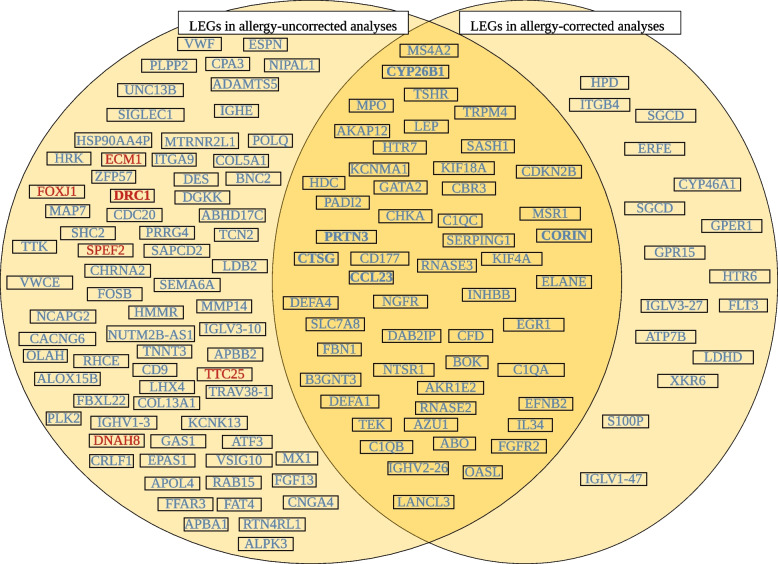
Distribution and Comparison of Leading Edge Genes Between Allergy-Corrected and Uncorrected Analyses. Figure shows LEGs with absolute mean log2 FC values over 0.5. Bold values, blue and red colours denote log2 FC > = 2, down- and upregulation, respectively. *Log2 FC* – Logarithm Twofold Change, *LEGs* – Leading Edge Genes

We considered two remaining phenotypic factors, allergy and female/male sex, that may have influenced our findings and their analysis could reveal interesting insights into MO. On one hand, correction for allergy—despite its overall beneficial effect on replicability—may have removed migraine-relevant genes from comparisons due to the high comorbidity and potential shared aetiology with migraine (38). We tested this possibility by comparing results of allergy-corrected and -uncorrected analyses. With the notable exception of 16 non-overlapping LEGs (Fig. 3) the majority of LEG69 and all LEG5 genes remained relevant in driving pathway level enrichments independent from allergy-correction. *CORIN* and *CYP26B1* remained LEGs with largest mean log2 FC, with the latter reaching FDR-significance at both time points in allergy-uncorrected analysis (Supplementary material 2,7,8,9).

### Sex-specific analyses confirm pathway-level findings and importance of CYP26B1 and CORIN

Correction for sex in above tests has ensured that the identified core results are important in both sexes. Nonetheless, sex-specific analyses are recommended in migraine research and migraine prevalence is two- to threefold in females compared to males [39]. Therefore, we speculated that analyses comparing the sexes may deliver additional insights about the stability of the found genes and pathways and can point to sex-specific characteristics for future studies.

Comparison of gene expression between female and male migraine patients with correction for control samples in the sexes left no significantly different genes at any time points neither in allergy-corrected nor in allergy-uncorrected analyses (Supplementary material 1), indicating lack of significant sex-specific gene-level differences.

Pathway-level findings indicated that the general comparison of migraine versus controls delivered stable results and pathway-level difference between the sexes stems from larger downregulation in men, and smaller downregulation or slight, non-significant upregulation in women (For full results see Supplementary material 2,7,8,9).

### Genetic associations of Leading Edge Genes refine background of expression changes

To test if the observed changes have an underlying genetic cause, we performed logistic regression analyses with age, sex, smoking status and history of allergy as covariates on SNPs of the LEG69 genes for migraine in the UK Biobank. No results survived Bonferroni-type multiple hypothesis correction (*p*-value < = 3.66 × 10^–6^, 0.05/13,650). Nominally significant hits were numerous and included variants from *EFNB2*, *CBR3*, *TEK*, *KCNMA1* and *CORIN*. Many SNPs below nominal threshold were related to *SGCD* (Supplementary material 10).

### CYP26B1 indicated a role for retinoic acid pathway in migraine

The fact that CYP26B1 is regulated by and metabolises all-trans-retinoic acid (atRA) and our previous results, i.e. 1) being the top gene with largest average log2 FC, 2) having stable, sex- and allergy-independent expression change and 3) showing no underlying SNP hits indicated that retinoic acid-related pathways may be involved in MO. Therefore, we conducted further investigations and first, scrutinized specific related gene sets in pathway-level results.

Allergy-corrected analyses showed a nominal significance for retinoic acid receptor signalling pathway at S2. This result became FDR-significant at S2 in allergy-uncorrected analysis, suggesting a potential involvement of retinoic acid signalling (Supplementary material 9).

### Genetic analyses of vitamin A pathway point to connection with LRP1

Logistic regression of vitamin A pathway SNPs for migraine with age, sex, history of allergy, smoking status and vitamin A intake correction was done in the UKB dataset.

Eleven polymorphisms, rs4759276, rs4759275, rs1385526, rs1799737, rs1466535, rs10876964, rs4759045, rs10876965, rs11172113, rs4367982, rs4759277 of the *LRP1* gene showed significant results for migraine with *p*-values below the multiple hypothesis correction threshold of 3.66 × 10^–6^ (0.05/13635, Supplementary material 11). Analyses correcting for sex, age, smoking, allergy and vitamin A showed that the associations are independent of vitamin A intake, excluding associations due to an underlying correlation of different vitamin A intake with migraine status and highlighting *LRP1* polymorphisms as potential independent causal factors. Seven *LRP1* SNPs that were significant in the UKB and were present in our population showed nominal significance for MO status independent of the corrections used (Supplementary material 11). In all tests conducted the minor alleles at the given positions showed protective effects against migraine, indicating risk for major allele carriers.

### Majority of genes behind pathway-level enrichment in migraine are regulated by RAR and RXR receptors

For further validation of an altered retinoic acid signalling raised by the GSEA results, regulatory connections between LEG69 and retinoic acid receptors *RARA, RARB, RARG, RXRA, RXRB, RXRG* were searched for in Gene Regulatory Network database (GRNdb) [40].

Results using bulk, healthy human data from GTEx resulted in 63.76% of the LEG69 being regulated by at least one of the receptors and 28 being regulated by multiple receptor families with the default NES threshold (> = 3). *CORIN* provided only one hit in pancreas tissue samples via *RXRA* (NES = 3.04, Supplementary material 12).

If pathological conditions were also included, 81.16% of LEG69 were regulated by at least one of the receptors with NES over 3 (Supplementary material 12).

### Migraine-associated drugs do not bind proteins of gene expression changes

To assess if migraine-associated compounds from DrugBank can act at the corresponding proteins of the identified LEG69, binding simulations with AutoDock Vina were run (since binding is a prerequisite of pharmacological effect). For LEG69, no migraine medications showed significant binding after multiple hypothesis correction (one-sided *p*-value < = 1.3617 × 10^–5^, Supplementary material 13).

### Binding predictions to proteins of gene expression changes find new drug candidates

Given the binding results for the gene expression changes and current migraine-associated compounds, we speculated that a search for potential drug candidates can help to find efficient therapies acting on the identified genes. Chemical structure-based machine learning prediction of drug-protein binding with HyperAttentionDTI showed that CYP26B1 is not bound by any investigated drug at the given significance level, while CORIN had four potential binding candidates with FDR < = 0.10 (Table 4, Supplementary material 14).

**Table 4:** Predicted drug candidates targeting CORIN in Migraine

Drug candidate	Predicted binding Z-score	FDR value for the Z-score	Literature reference, if any (PMIDs)
probucol	2.570	0.070	10601122
fasoracetam	2.557	0.073	
ginsenosides	2.454	0.081	1648425, 3248333, 32420095
rovafovir	2.454	0.098	

Table shows four potential drug candidates, predicted binding scores and FDR values from 11,164 drugs in DrugBank with predicted FDR significant (FDR < = 0.1) binding predictions to *CORIN*. Last column indicates PMIDs, with reference for the given substances, if any. *Abbreviations: FDR* – False Discovery Rate, *PMIDs – *PubMed IDs

Estimating gene expression-weighted pathway-level binding showed numerous potential binding candidates (a selected list with *p*-values below 0.0005 can be found in Supplementary material 15). We have to note, the list of gene- and pathway-level candidates includes both (potential) migraine-promoting and -treating drugs, since the method is based on binding predictions, not direction of effects. Among the significant (*p* < = 0.0005) substances, several drugs showed a connection to migraine, migraine symptoms, headaches and -treatments in the literature (see Table 5). In addition, several, already known migraine-associated compounds (in DrugBank, bold in Table 5) were found among pathway-level drugs, like eletriptan.

**Table 5:** Potential drug candidates and associated pathways for migraine therapy

Drugs	Associated pathway(s)	Reference(s) (PMIDs)
**buclizine**	Positive regulation of cell death, response to lipid, transmembrane receptor protein tyrosine kinase signalling pathway	22469258
**mecloxamine**	Positive regulation of cell death	17373440,9265005,8366753,6342291
**verapamil**	Positive regulation of cell death	2668225
tetrandrine	Positive regulation of cell death	29147842
olmesartan	Transmembrane receptor protein tyrosine kinase signaling pathway	16618270,16942482,31515634
nicardipine	Cytokine mediated signaling pathway, innate immune response, phagocytosis, positive regulation of cell death, transmembrane receptor protein tyrosine kinase signaling pathway	30600979,2245455
tubocurarine	Positive regulation of cell death, transmembrane receptor protein tyrosine kinase signaling pathway	28496430,10686170
zafirlukast	Positive regulation of cell death	17691939,15648777,10759916
opc-28326	Positive regulation of cell death	17103145
indoramin	Positive regulation of cell death	49624,324566
nystatin	Positive regulation of cell death	28041915
pasireotide	Positive regulation of cell death	29925553
thioridazine	Transmembrane receptor protein tyrosine kinase signaling pathway	10667670
bicuculline	Positive regulation of cell death	36259130,12499053
**bi 44370 ta**	Positive regulation of cell death	32525262
berberine	Transmembrane receptor protein tyrosine kinase signaling pathway	23758551
resiniferatoxin	Positive regulation of cell death	29187670,23155193
ondansetron	Positive regulation of cell death	32433024,20661681
**lomerizine**	Transmembrane receptor protein tyrosine kinase signaling pathway	37194515,29221971
**eletriptan**	Positive regulation of cell death	15853473

The table shows 1) selected migraine-associated drugs (bold) and potential drug candidates with significant (*p*-value < = 0.0005) weighted binding to significantly replicated pathways between migraine and controls and 2) supporting literature references. See also Supplementary material 15. *Abbreviations*: *PMIDs* – PubMed IDs

## Discussion

Previous transcriptomic investigations in MO yielded inconsistent findings and remained detached from existing migraine pathophysiology theories, genetic findings and the method’s potential for drug development (5–10). Here, we report replicated pathway-level gene expression changes in MO at interictal state obtained after correction for underlying masking phenotypic differences, allergy and sex. The results highlight two genes, *CORIN* and *CYP26B1* and downregulated metabolic, cardiovascular, immunologic-, oxidative stress- and hormone regulation-related pathways as important transcriptomic findings. We show that genetic polymorphisms cannot explain the observed *CYP26B1* downregulation, and provide consistent proofs for its marker role of a dysregulated retinoic acid receptor signalling in patients and its potential underlying cause, reduced retinoic acid availability due to disease-associated genetic polymorphisms within the *LRP1* gene. We also show that few current migraine medications utilise the corresponding proteins of the observed transcriptomic alterations, and highlight gene- and pathway-level drug candidates, like probucol, tetrandrine and indoramin to promote future drug development.

No significantly replicated gene for MO could be found in our core analysis. This was in part due to the replication criterion, partly due to allergy-correction, as evidenced by the large number of additional pathways and the significantly replicated *CYP26B1* gene in the allergy-uncorrected analysis. Another reason may have been the relatively smaller change of expression of individual genes, corresponding to the polygenic nature of MO [41], making detection of gene-level differences harder. The latter can be compensated, at least in part, by pathway-level analysis methods.

Indeed, numerous replicable results emerged with pathway-level analyses and these aligned to existing migraine pathophysiology theories: downregulated and altered amino acid-, lipid- and carbohydrate metabolism [42–45], immunologic processes [8, 46], cardiovascular comorbidities [38, 47] and hormonal influences [48] have been implicated in MO previously. It remains uncertain, if in contrast to our interictal study, during a migraine attack different findings would have emerged (as suggested by ref. [6]). Nonetheless, agreement of the observed changes with numerous proposed attack mechanisms in the literature [8, 38, 42–48] suggests that our findings may not only represent interictal characteristics, but are more general. Future studies need to decipher if the present results correspond only to interictal state and if during migraine attack new gene expression changes (e.g., other pathways) emerge or expression level of the found pathways change.

Sex- and allergy-dependent analyses indicated a promising direction for future studies investigating MO-relevant pathways. The more than 3-times larger number of significantly replicated pathways and the additional genes in allergy-uncorrected analysis clearly indicated that allergy may have common mechanisms with MO, while analyses directly comparing the two sexes suggested that male-specific pathways that were also significant in females were found by general migraine versus control comparison confirming the stability of our findings, but leaving room for, especially, male-specific studies for the future.

**Fig. 4:**
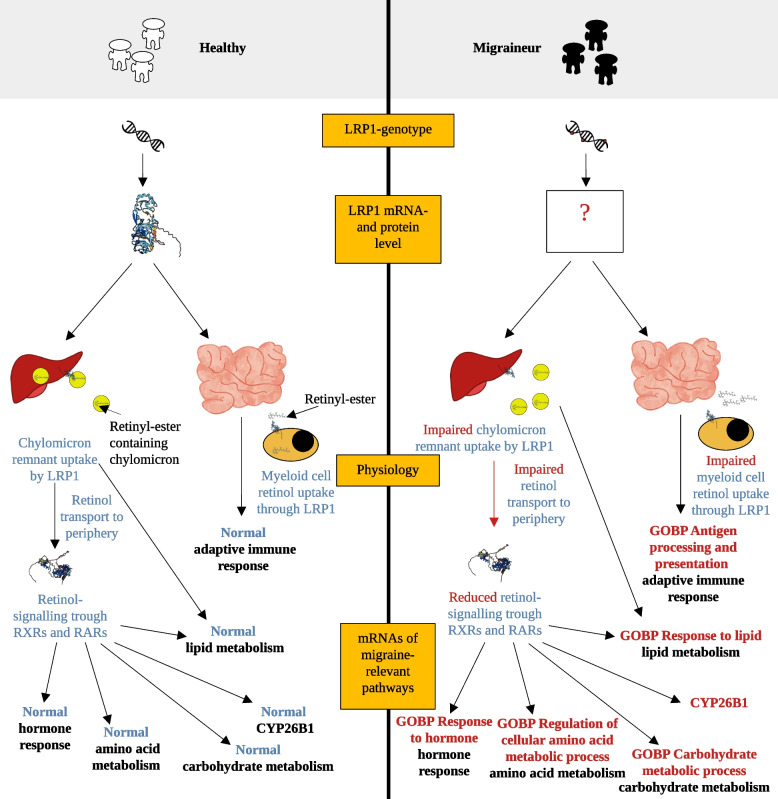
Proposed Mechanistic Model: Role of Vitamin A Signaling in Migraine Pathogenesis. Figure depicts how LRP1 polymorphisms, impacting LRP1 gene expression (not measured due to low blood-expression) and protein levels, may lead to migraine-related transcriptomic changes. Key processes involve retinyl-ester and retinol uptake in hepatocytes and myeloid cells. The changes ultimately result in the downregulation of CYP26B1 in a replicated allergy-uncorrected analysis, indicative of underlying physiological alterations, and other downregulated migraine-related pathways, where one example pathway each has been mentioned. Measured changes are highlighted in bold black fonts

Among sex- and allergy-independent migraine-associated genes, *CYP26B1* and *CORIN* deserve particular attention. Both 1) the downregulated *CYP26B1*-levels, which are reliant on the substrate, all-trans retinoic acid (atRA) in the heart and vasculature [49–51] and 2) the lack of *CYP26B1* SNPs associated with migraine even on a nominal level, indicated a marker role for *CYP26B1* for reduced atRA levels in MO. Analyses of vitamin A pathway SNPs implicated *LRP1* polymorphisms as potential underlying cause for reduction in *CYP26B1* levels. LRP1 helps retinyl-ester containing chylomicron remnant uptake [28] in the liver, from where vitamin A derivatives are transported to the periphery [28] LRP1 is also involved in the uptake of serum amyloid A-retinol complexes in immune cells of the intestine [52]. Thus, LRP1 is capable of influencing both direct retinol uptake into immune cells and peripheral atRA abundance available for signaling (Fig. 4). Impairment of the latter processes naturally can lead to decreased levels of hepatic- and peripheral retinoic acid derivatives and result in diminished atRA-dependent signalling and *CYP26B1* expression.

RXR/RAR receptors are key players in atRA-dependent signaling and known to influence gene expression. The found regulatory connections between 63.76% of LEGs and RXR/RAR receptors show how a reduced retinoic acid level can be connected to the LEGs and pathway-level changes. In agreement, pathway-level downregulations including amino acid- and carbohydrate metabolism and oxidative stress can also be related to reduced atRA levels, as vitamin A deficiency could induce growth restriction and reduced gluconeogenesis, glycolysis, and likely, protein catabolism in the liver [53] and a reduced antioxidant response [54]. Furthermore, LRP1-dependent reduced retinol uptake may be the cause for downregulation in immune system-related pathways by restricting retinol availability for immune cells [52], while impaired chylomicron remnant uptake [28] may be responsible for the observed downregulated lipid metabolism and elevated lipid availability in the periphery, an often comorbid condition with migraine.

All in all, *CYP26B1* downregulation together with the genetic polymorphisms of *LRP1*, the regulatory connections between LEGs and RXR/RAR receptors and several downregulated pathway categories paint a consistent picture about a reduced retinoic acid signaling in MO. It extends previous meta-analyses [3, 4], which found consistent associations between *LRP1* polymorphisms and migraine, by providing a genetically founded mechanism for the transcriptomic and pathophysiological changes, which were independent of the different vitamin A intake between migraine patients and controls. The proposed mechanism also establishes a connection between migraine subtypes, since 1) meta-analysis showed relevance for *LRP1* lead variant for both MO and migraine with aura (4) and 2) reduced retinol binding protein 4, a protein with high sensitivity and specificity to measure vitamin A deficiency, was found in both subtypes [55]. In sum, the found changes 1) integrate alterations in various pathways corresponding to migraine pathophysiology theories via an altered retinoic acid signaling, 2) and raise the possibility of therapeutic approaches targeting this pathway in MO in clinical settings.

*CORIN* downregulation seemed to be an independent alteration in MO, as regulatory connections between retinoic acid receptors and *CORIN* were scarce. CORIN is a key enzyme in atrial and brain-derived natriuretic peptide (ANP, BNP, respectively) synthesis [56]. Reduced level of CORIN may explain for 1) a report about decreased blood ANP levels in migraine [57] and 2) increased BNP precursors in patients [58]. Furthermore, reduced BNP level, a likely result of downregulated *CORIN* in our study, has been shown to mimic effects of a key mutation in the familial form of migraine on P2X3 receptors in trigeminal neurons and to facilitate trigeminal sensitization, the primary cause for migraine pain [59, 60].

Additional LEGs of interest also emerged. The overall picture of these candidates (Fig. 2) shows that the relationship between genetic background and observed transcriptomic changes in MO is complex. As Bonferroni-correction is known to filter positive findings in favor to control type 1 errors [61–63], some genes, such as *EFNB2*, *CBR3*, *TEK*, *KCNMA1*, *HTR7* with nominally significant SNPs and connection to migraine or its characteristics [64–68], may (also) have genetically determined changes in their gene expression. Another gene of interest may be *SGCD*, which is involved in cardiomyopathy and age-related macular degeneration [69], and provided a large majority of SNP-level nominally significant hits, but remained unassociated previously with MO. These findings propose that different underlying mechanisms are reflected in the current findings: 1) with some directly linked to underlying SNPs and 2) some connected indirectly, via probable, functional, secondary links.

Despite replicable, consistent findings on gene- and pathway-levels, existing migraine medications showed limited binding to corresponding proteins in accordance with their significant, but modest absolute therapeutic benefit [70]. This is not due to non-existing chemical agents to manipulate these targets. Multiple drug candidates from Drugbank were shown to bind LEGs or significant pathways. In fact, some of these have been tested for migraine or its symptoms already, like probucol, ginsenosides, nicardipin, tetrandrine (Tables 4–5). These may serve as templates for future drug developments. At the same time, results contain several hundred additional small molecule compounds predicted to act at the identified LEGs and pathways. These, especially, if combined with expert knowledge and well-formulated hypotheses, can deliver more efficient MO medications in the future.

Our approach comes with limitations. First, the use of whole blood samples was limited in identifying differentially expressed genes with low or no absolute expression in blood (like *LRP1*). It has to be noted, however, that 1) the reported *CYP26B1* and *CORIN* downregulation and metabolic, immunologic, cardiovascular and hormonal pathways’s effects have mostly tissue-independent implications, 2) expression from blood samples correlated well with e.g., that of brain samples especially, when relevant genes were examined [71], 3) genetic analyses by nature report tissue-independent associations. Second, single-cell gene expression may have provided detailed results, but our intention in the present study was not a detailed temporal and spatial resolution of cell-type specific changes. Third, we did not measure protein levels, thus, it cannot be excluded that some gene expression changes do not manifest in protein level changes. However, this is unlikely to substantially influence our conclusions, since mRNA and protein levels show reasonable correlation with divergence often attributed to post-transcriptional regulation [72], a phenomenon less relevant in case of mRNA downregulations. Fourth, we could not directly measure retinoic acid/vitamin A levels in our small sample due to its likely degradation at the time we realised its importance. Few subjects also reported taking vitamin A containing supplements in the control group (Supplementary material 1) with variable amounts and uncertain regimen. Despite these uncertainties, we conducted confirmatory analyses with corrections for vitamin A containing supplement intake to test the independence of our results. Results have shown marginal changes in log2 FC and significance values of key findings (Supplementary material 16), indicating that our core results are independent from supplement intake. Furthermore, vitamin A deficiency is a rare condition in the examined population [73] and the chance that such individuals were accidentally assigned disproportionately to the MO group is improbable. Fifth, we did not correct for contraceptive use due to the various active ingredients used by the subjects (Supplementary material 1). Sixth, one participant took an anti-allergic medication at the second time point. The latter two limitations may have contributed to heterogeneity of findings, but for this very reason were unlikely to influence those that remained significant at both time points. Seventh, additional factors, like diet, sleep and exercise may have influenced our results, for which, however, we had less reliable data. However, for the only factor showing significant differences between MO and controls, a measure of sleep quality (Supplementary material 1), we conducted confirmatory analyses. Similarly to vitamin A containing supplement intake, results showed that inclusion of sleep quality as co-variate only slightly changed log2 FC and significance values (Supplementary material 16). Eighth, genomic analyses use Bonferroni correction as standard despite the potential for false negatives, therefore, nominally significant results in genetic analyses may be potential candidates for future studies to test. Ninth, CYP26B1 protein levels have not been validated by further experiments and the connection between LRP1 and CYP26B1 remains hypothetical, albeit with strong empirical evidence for both being involved in retinoic acid signaling. The above are unlikely to severely impact the interpretability or generalizability of the present findings due to the study’s rigorous selection criteria, methodological detail and replication criterion., Future studies compensating these, nonetheless, could definitely add further insights.

## Conclusion

All in all, the presented results provide solid support for existing pathophysiology theories through the stable, interictal transcriptomic map of MO, which provided both gene- and pathway level candidates for the underlying pathophysiology. In addition, our study also delivered drug candidates and interventions to manipulate these MO-associated gene- and pathway level targets. Furthermore, the present study provided consistent evidences for a factor behind a share of previous pathological observations in metabolic-, immunologic- and cardiovascular processes in migraine in the form of an altered retinoic acid signaling and implicitly suggests that targeting elements of this pathway in patients may alleviate some of the previously found pathological alterations in the disorder.

## Supplementary Information

Supplementary Material 1

Supplementary Material 2

Supplementary Material 3

Supplementary Material 4

Supplementary Material 5

Supplementary Material 6

Supplementary Material 7

Supplementary Material 8

Supplementary Material 9

Supplementary Material 10

Supplementary Material 11

Supplementary Material 12

Supplementary Material 13

Supplementary Material 14

Supplementary Material 15

Supplementary Material 16

## Data Availability

The RNA-Seq datasets generated and analysed during the current study are available in the ArrayExpress repository, https://www.ebi.ac.uk/biostudies/arrayexpress/studies/E-MTAB- 13397.

## Abbreviations

atRA: All-trans Retinoic Acid
BP: Biological Processes
CNN: Convolutional Neural Network
FDR: False Discovery Rate
GO: Gene Ontology
GRNdb: Gene Regulatory Network database
GSEA: Gene Set Enrichment Analysis
LEGs: Leading Edge Genes
LEG5: Leading Edge Genes with an absolute mean logarithm twofold change value > = 2
LEG69: Leading Edge Genes with an absolute mean logarithm twofold change value > = 0.5
log2 FC: Logarithm twofold change
MF: Molecular Function
MIDAS: Migraine Disability Assessment
MINI: Mini International Neuropsychiatric Interview
MO: Migraine without Aura
MSigDB: Molecular Signature Database
NES: Normalized Enrichment Score
RNA-Seq: MRNA Sequencing
S1: Time Point 1
S2: Time Point 2
SGCD: Sarcoglycan Delta
SNP: Single Nucleotide Polymorphism
UKB: UK Biobank
QC: Quality control
