# Supplementary material for: The interictal transcriptomic map of migraine without aura
Source: J Headache Pain. 2025 May 12;26(1):109. doi: 10.1186/s10194-025-02033-z (PMC12067696; doi:10.1186/s10194-025-02033-z)
Supplement: Supplementary file 1 — Supplementary Material 1 [file 10194_2025_2033_MOESM1_ESM.docx]

**Supplementary Information**

The interictal transcriptomic map of migraine without aura

Peter Petschner†^1,2,3,4^, Sahel Kumar†^2^, Duc Anh Nguyen^1,5^, Dora Torok^2,3^, Zsofia Gal^2,3^, Daniel Baksa^2,3,6^, Kinga Gecse^2,3^, Gyongyi Kokonyei^2,3,7^, Hiroshi Mamitsuka^1^, Gabriella Juhasz*^2,3^

^1^Bioinformatics Center, Institute of Chemical Research, Kyoto University; Uji, Kyoto, Japan.

^2^Department of Pharmacodynamics, Faculty of Pharmaceutical Sciences, Semmelweis University; Budapest, Hungary.

^3^NAP3.0-SE Neuropsychopharmacology Research Group, Hungarian Brain Research Program, Semmelweis University; Budapest, Hungary.

^4^Research Unit for Realization of Sustainable Society, Kyoto University; Uji, Kyoto, Japan.

^5^Hanoi University of Science and Technology; Hanoi, Vietnam.

^6^Department of Personality and Clinical Psychology, Institute of Psychology, Pazmany Peter Catholic University; Budapest, Hungary.

^7^Institute of Psychology, ELTE Eötvös Loránd University; Budapest, Hungary.

*Corresponding author: Gabriella Juhasz, Nagyvarad ter 4, 1089, Budapest, Hungary, +36-1-210-4411, [juhasz.gabriella@semmelweis.hu](mailto:juhasz.gabriella@semmelweis.hu)

† these authors contributed equally to the present work

**Contents**

1. **Supplementary results**
   1. **Biological coefficient of variation plots**
      1. Figure 1. BCV plot at S1 of migraine vs control comparisons
      2. Figure 2. BCV plot at S1 of male migraineur vs male control comparison
      3. Figure 3. BCV plot at S1 of female migraineur vs female control comparison
      4. Figure 4. BCV plot at S1 of female migraineur vs male migraineur comparison
      5. Figure 5. BCV plot at S2 of migraineur vs control comparison
      6. Figure 6. BCV plot at S2 of male migraineur vs male control comparison
      7. Figure 7. BCV plot at S2 of female migraineur vs female control comparison
      8. Figure 8. BCV plot at S2 of female migraineur vs male migraineur comparison
   2. **Descriptive statistics of the migraine transcriptomic cohort**
      1. Table 1. Population descriptors of transcriptomic cohort – sleep quality/chronotype, diet, and exercise
      2. Table 2. Population descriptors of transcriptomic cohort - history of allergy
      3. Table 3. Population descriptors of transcriptomic cohort - smoking status
      4. Table 4. Population descriptors of transcriptomic cohort - supplement intake
      5. Table 5. Population descriptors of transcriptomic cohort - contraceptive use
   3. **Descriptive statistics of the additional cohorts used in the study**
      1. Table 6. UK Biobank sample characteristics
      2. Table 7. Migraine transcriptomic cohort sample characteristics
      3. Table 8. Results of the comparison of retinol and vitamin A retinol equivalent intake between migraine and control group
   4. **Results of allergy- and sex dependent analyses**
2. **Supplementary methods**
   1. **Phenotypic characterization of the transcriptomic and migraine cohort**
   2. **Power Calculations**
      1. Figure 9. Power estimate versus mean count plot
   3. **Sequencing and sequencing data analysis**
   4. **Genomic data analysis**
   5. **AutoDock Vina and machine learning-based predictions**
      1. Figure 10. Schematic workflow of AutoDock Vina binding calculations between drugs and genes
      2. Figure 11. Schematic workflow of machine learning based drug-protein binding predictions
3. **Supplementary References**

**Biological coefficient of variation plots**

In RNA sequencing (RNASeq) experiments, the biological coefficient of variation (BCV) is a statistical measure used to assess the variability in gene expression levels between biological replicates. It quantifies the extent of biological variability, which refers to the natural differences that occur among individual samples or organisms.

Plots in **Figures 1-8** show levels of BCV and were not considered outliers by visual inspection.


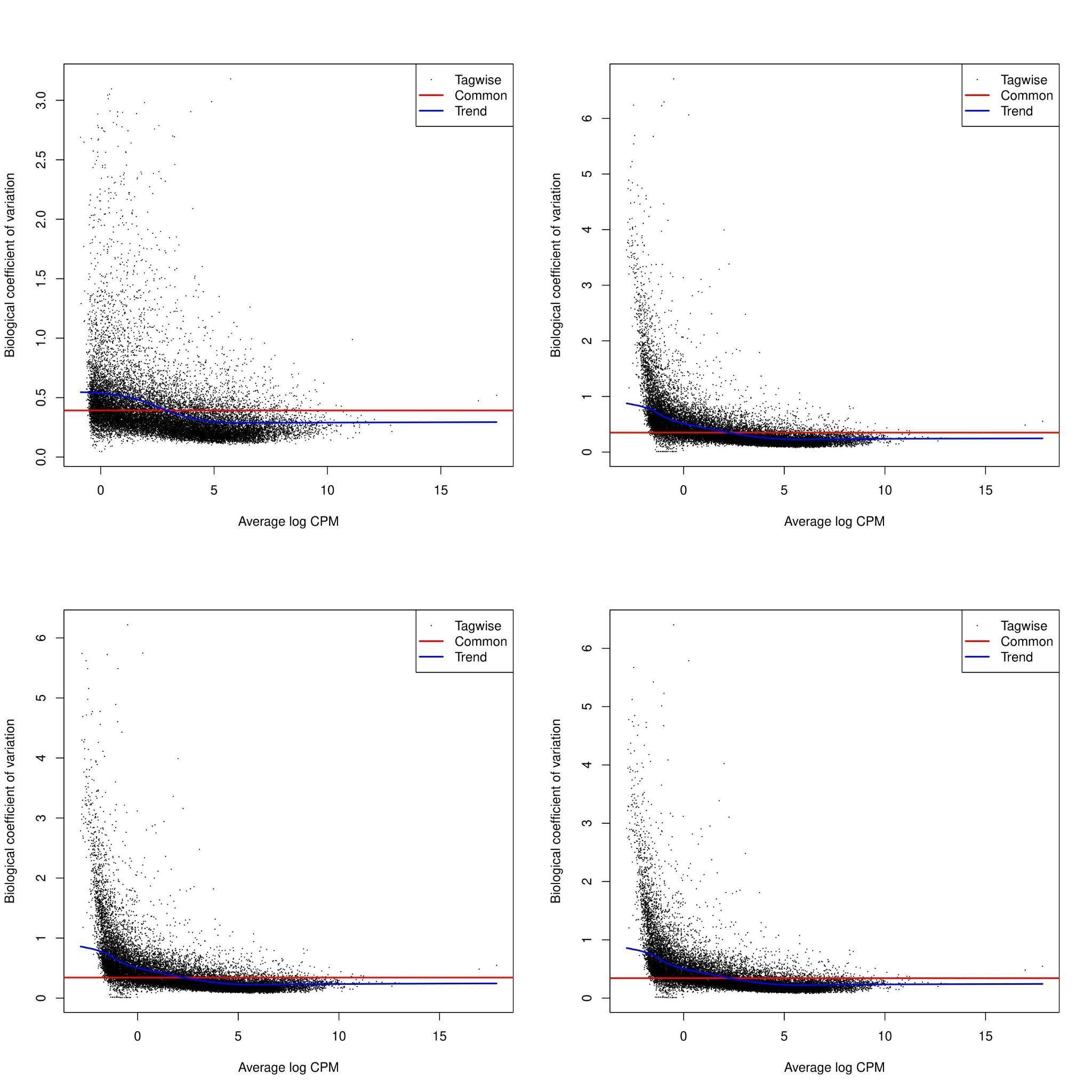


**Figure 1. BCV plot at S1 of migraine vs control comparisons**

*Upper left* figure represents data just for Age+Sex correction, *upper right* figure shows Age+Sex+Allergy correction, *lower left* figure has the Age+Sex+Smoking correction and *lower right* corner highlights the Age+Sex+Allergy+Smoking correction. Naming conventions correspond to main text.


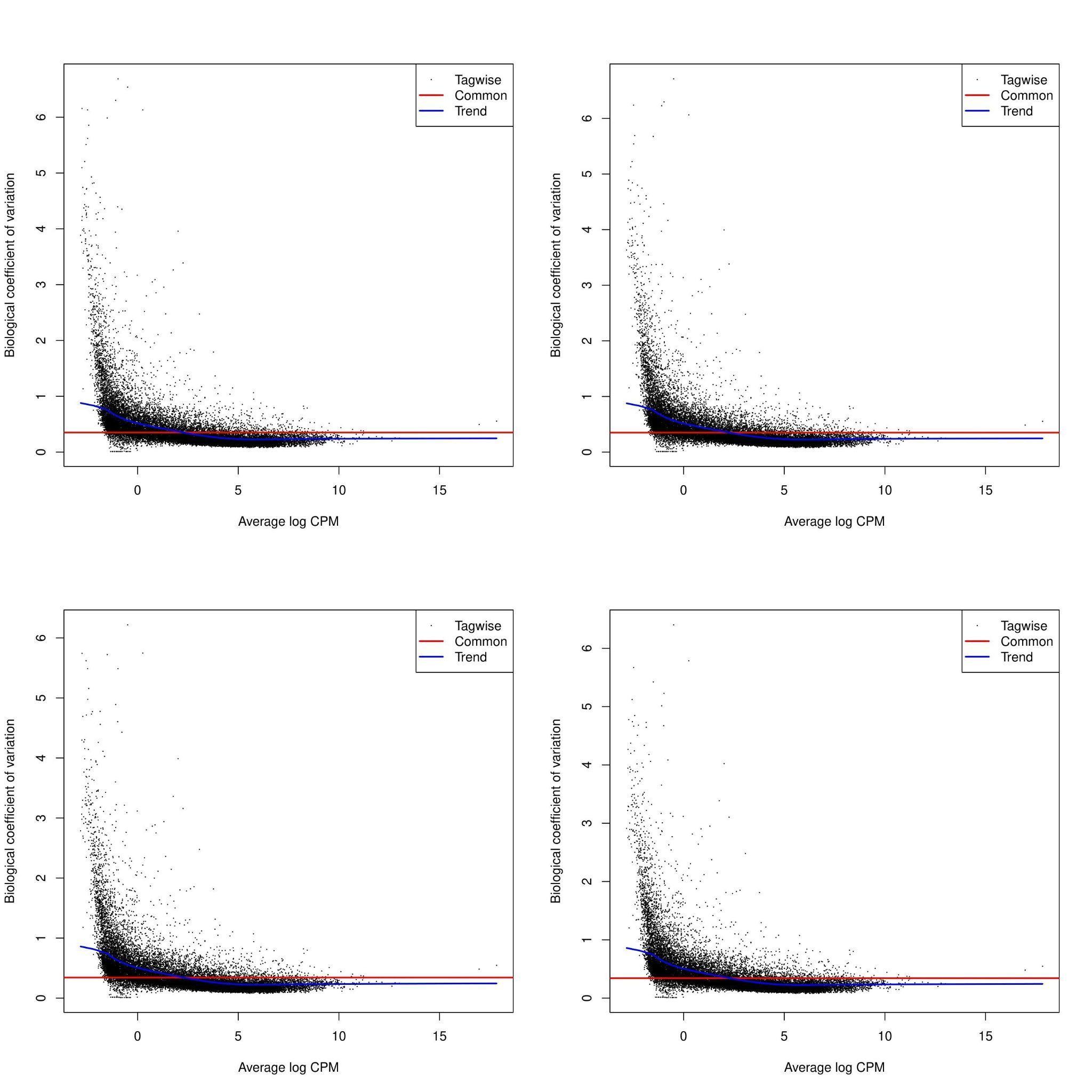


**Figure 2. BCV plot at S1 of male migraine** **vs male control comparison**

*Upper left* figure represents data just for Age correction, *upper right* figure shows Age+Allergy correction, *lower left* figure has the Age+Smoking correction and *lower right* corner highlights the Age+Allergy+Smoking correction.


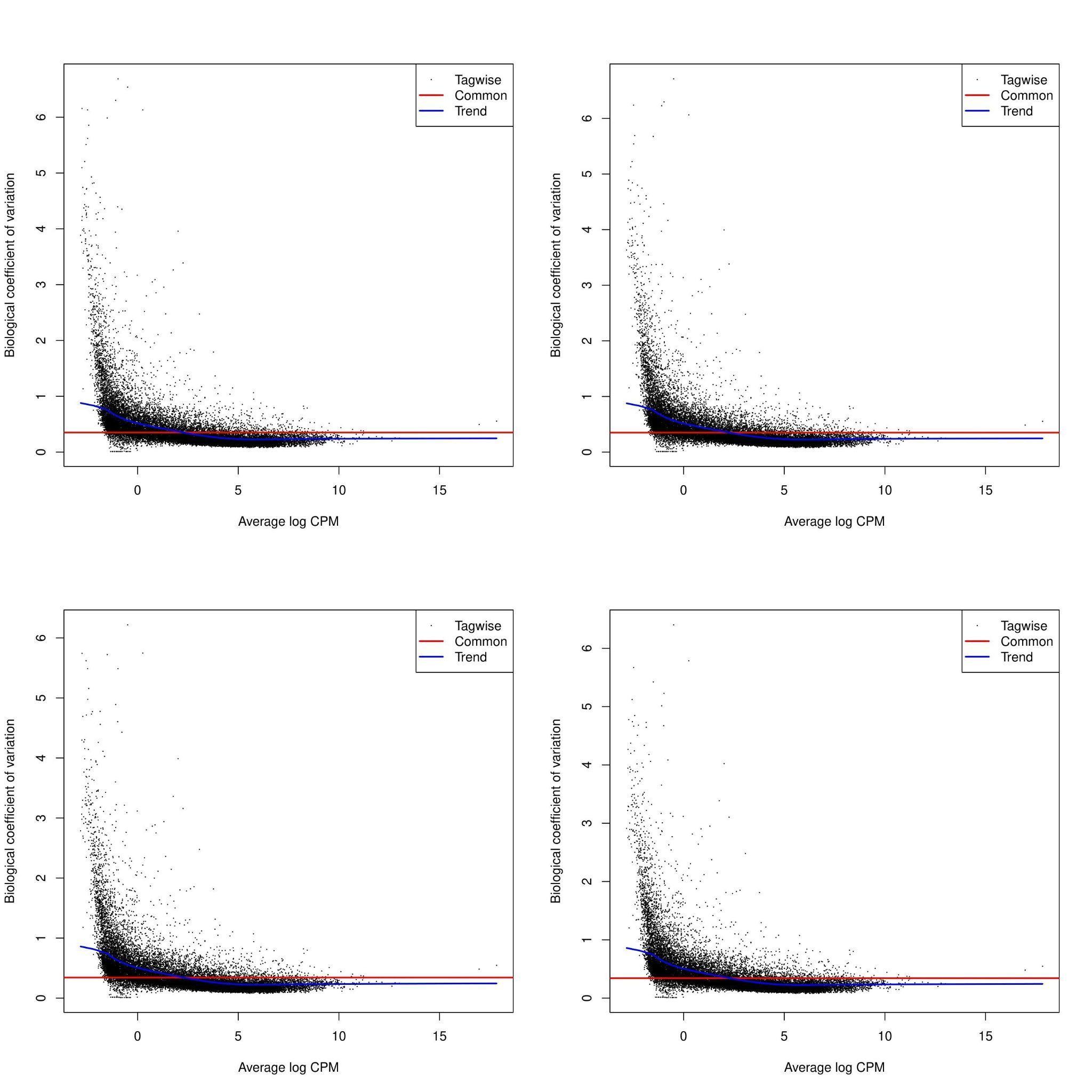


**Figure 3. BCV plot at S1 of female migraine vs female control comparison**

*Upper left* figure represents data just for Age correction, *upper right* figure shows Age+Allergy correction, *lower left* figure has the Age+Smoking correction and *lower right* corner highlights the Age+Allergy+Smoking correction.


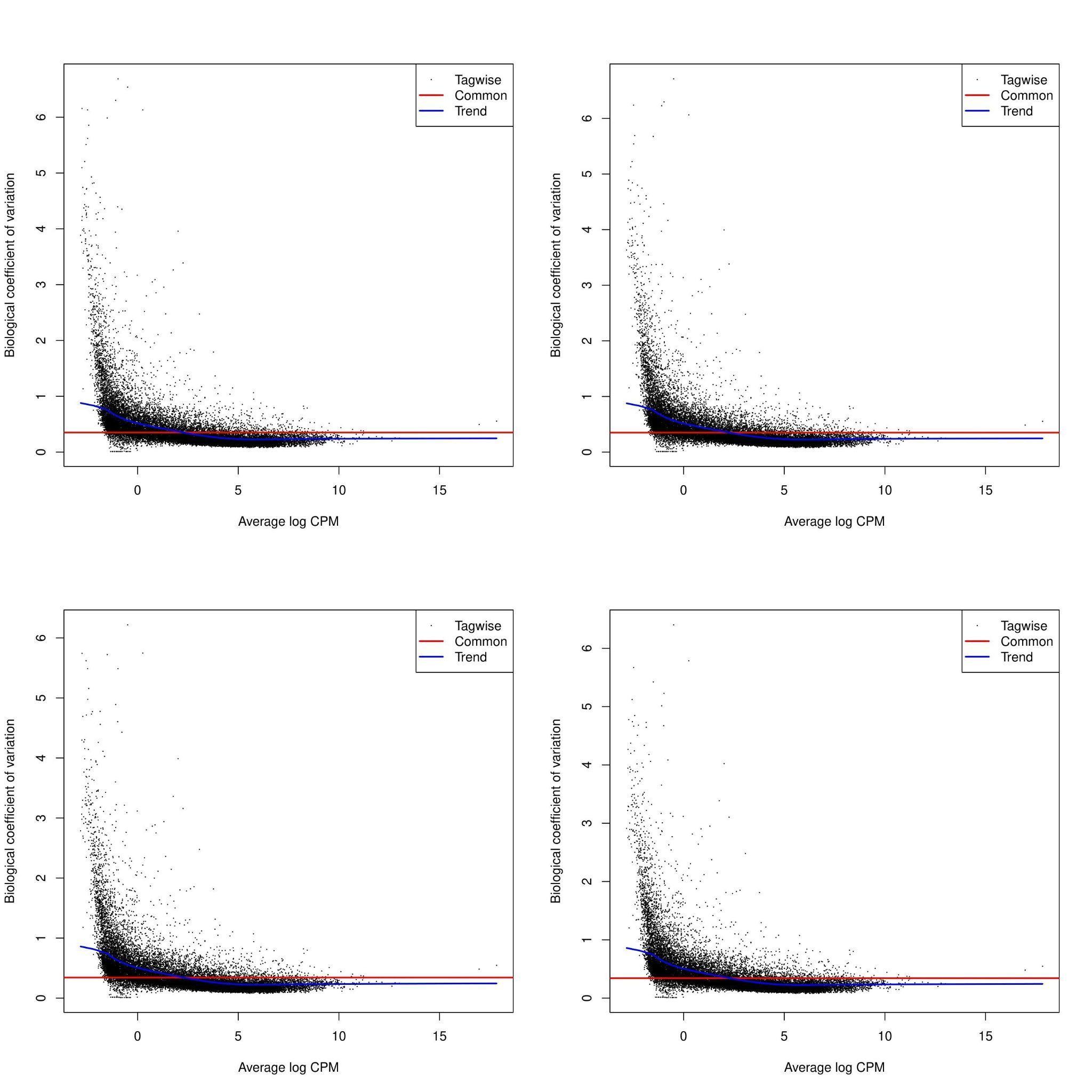


**Figure 4. BCV plot at S1 of female migraine vs male migraine comparison**

*Upper left* figure represents data just for Age correction, *upper right* figure shows Age+Allergy correction, *lower left* figure has the Age+Smoking correction and *lower right* corner highlights the Age+Allergy+Smoking correction.


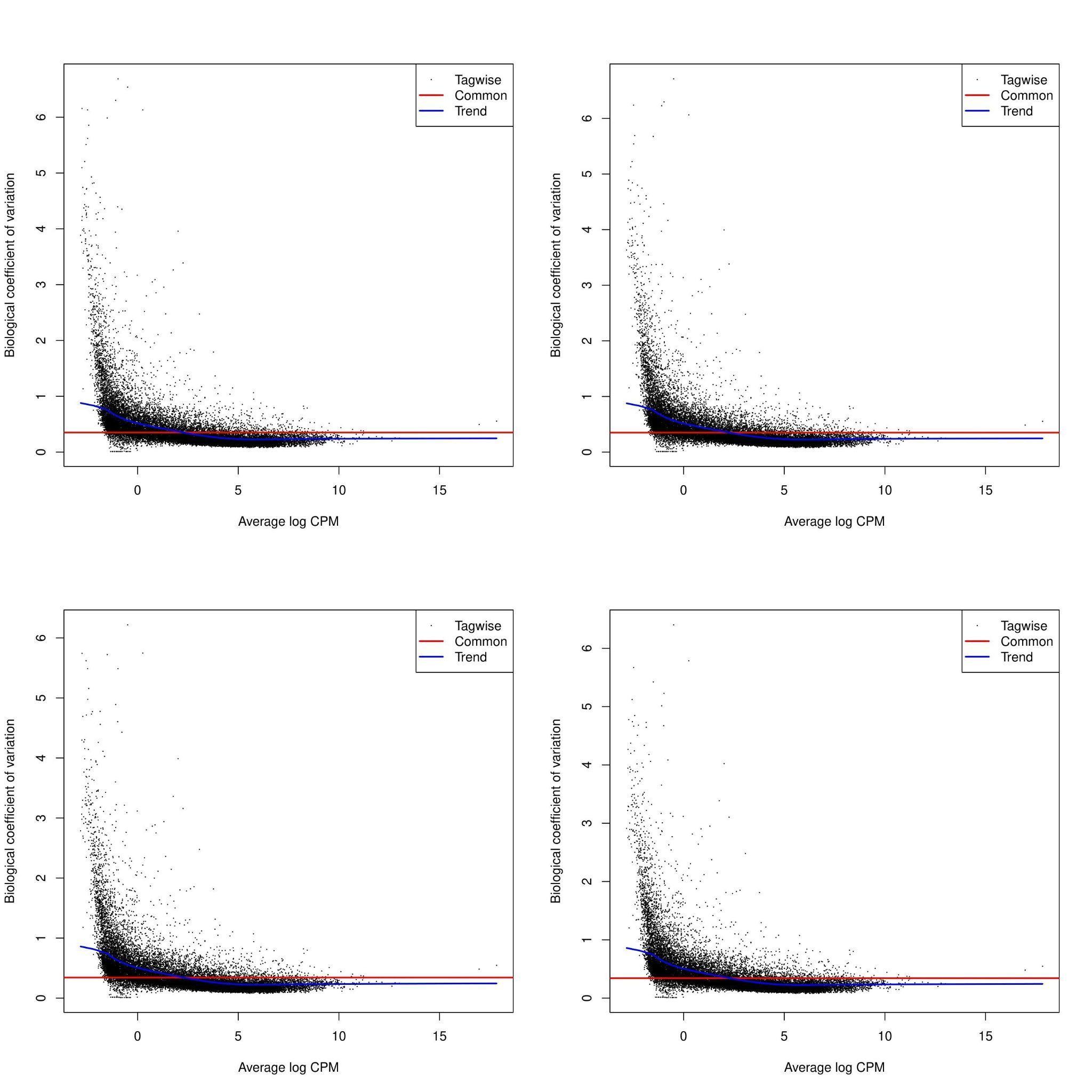


**Figure 5. BCV plot at S2 of migraine vs control comparison**

*Upper left* figure represents data just for Age+Sex correction, *upper right* figure shows Age+Sex+Allergy correction, *lower left* figure has the Age+Sex+Smoking correction and *lower right* corner highlights the Age+Sex+Allergy+Smoking correction.


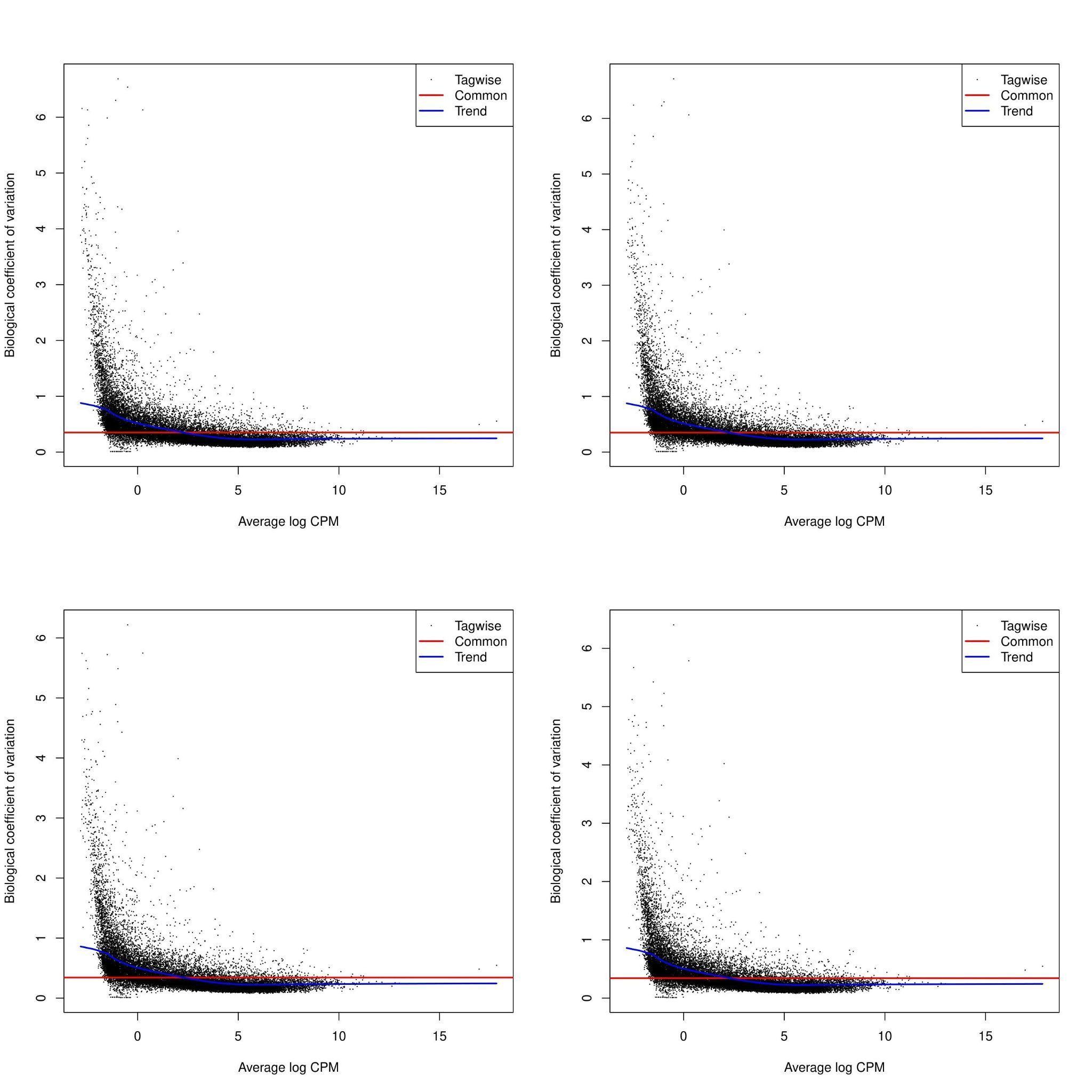


**Figure 6. BCV plot at S2 of male migraine vs male control comparison**

*Upper left* figure represents data just for Age correction, *upper right* figure shows Age+Allergy correction, *lower left* figure has the Age+Smoking correction and *lower right* corner highlights the Age+Allergy+Smoking correction.


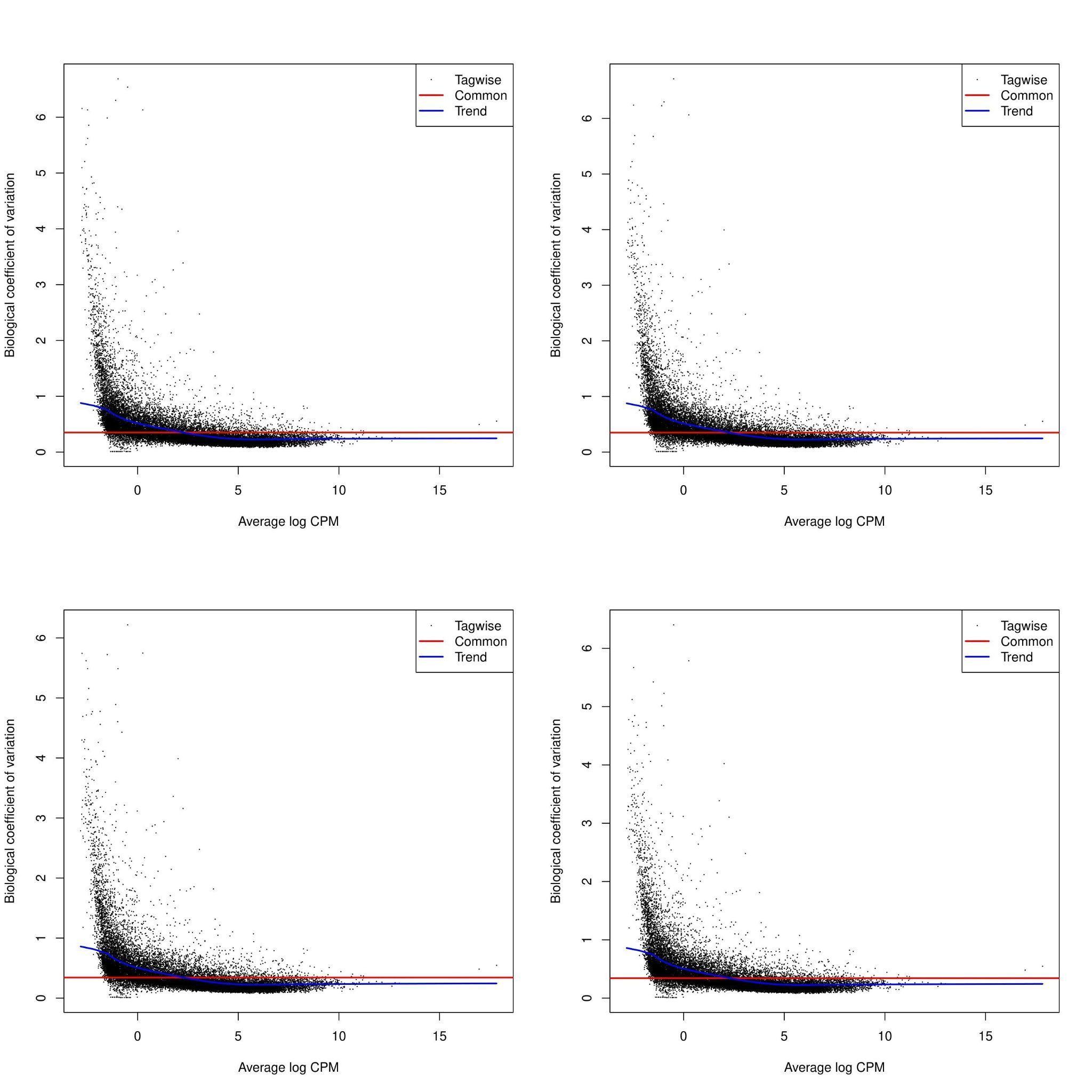


**Figure 7. BCV plot at S2 of female migraine vs female control comparison**

*Upper left* figure represents data just for Age correction, *upper right* figure shows Age+Allergy correction, *lower left* figure has the Age+Smoking correction and *lower right* corner highlights the Age+Allergy+Smoking correction.


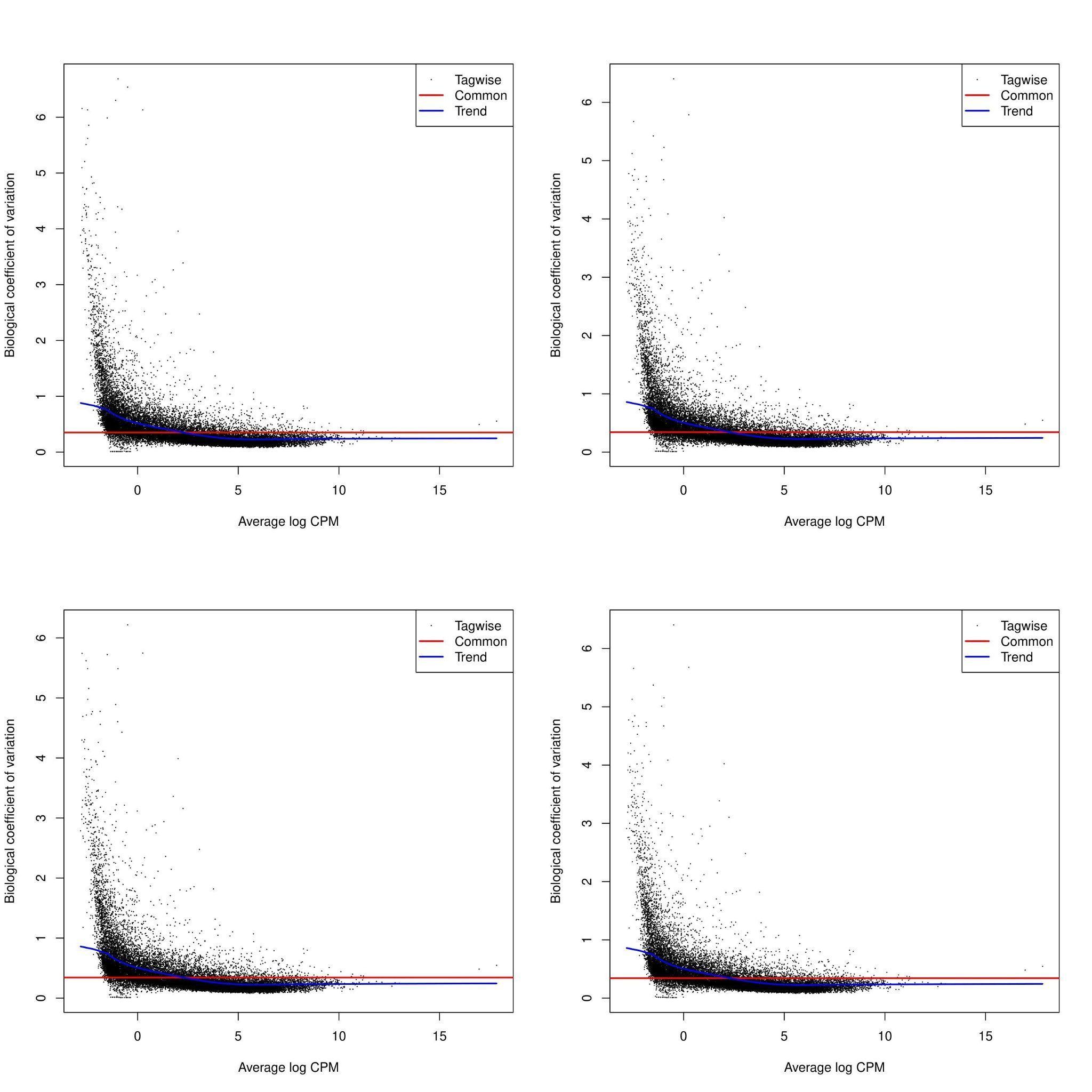


**Figure 8. BCV plot at S2 of female migraine vs male migraine comparison**

*Upper left* figure represents data just for Age correction, *upper right* figure shows Age+Allergy correction, *lower left* figure has the Age+Smoking correction and *lower right* corner highlights the Age+Allergy+Smoking correction.

**Descriptive statistics of the migraine transcriptomic cohort**

In the following detailed descriptive statistics are visible of the transcriptomic cohort and, where appropriate X^2^ or Mann-Whitney U test results are also presented to assess significance of differences.

# **Table 1. Population descriptors of the transcriptomic cohort – sleep quality/chronotype, diet, and exercise**

| Variable | | Question | | Response Options Con(Mig) | | n= Con(Mig) | | Test Statistic | | p-value |  |
| --- | --- | --- | --- | --- | --- | --- | --- | --- | --- | --- | --- |
| Sleep Quality | Do you have trouble falling asleep or do you wake up in the middle of the night? | |  | Never/Rarely – 21(7) | | | 29(22) | U = 192.5 | | **0.007012** | |
|  |  |  |  | Sometimes – 6(12) | | |  |  |  |  |  |
|  |  |  |  | Often/Often – 2(3) | | |  |  |  |  |  |
| Sleep Chronotype | Do you consider yourself a morning or evening person? | | 1 | Definitely morning – 3(2) | | | 27(22) | U = 282 | | 0.7595 | |
|  |  |  | 2 | More morning than evening – 7(4) | | |  |  |  |  |  |
|  |  |  | 3 | More evening than morning– 9(10) | | |  |  |  |  |  |
|  |  |  | 4 | Definitely evening – 8(6) | | |  |  |  |  |  |
|  |  |  | 5 | Don't know – 2(0) | | |  |  |  |  |  |
| Diet | | Do you eat vegetables regularly? | | Yes – 26(21) | | 29(22) | | χ² = 0.056 | | 0.8126 |  |
|  |  |  |  | No – 3(1) | |  |  |  |  |  |  |
| Diet | | Do you regularly eat fruit? | | Yes – 24(20) | | 29(22) | | χ² = 0.182 | | 0.6694 |  |
|  |  |  |  | No – 5(2) | |  |  |  |  |  |  |
| Exercise | | How often do you exercise? | | 1 | Daily – 5(2) | 29(22) | | U = 389 | | 0.1653 |  |
|  |  |  |  | 2 | 2–3×/week – 13(8) |  |  |  |  |  |  |
|  |  |  |  | 3 | 4–5×/month – 6(4) |  |  |  |  |  |  |
|  |  |  |  | 4 | Occasionally – 4(8) |  |  |  |  |  |  |
|  |  |  |  | 5 | Never – 1(0) |  |  |  |  |  |  |

Group-wise distribution of responses related to sleep quality/chronotype, dietary habits, and physical activity in Mig and Con. Mann-Whitney U tests were used for ordinal variables (sleep and exercise), and Chi-squared tests for categorical variables (vegetable and fruit consumption). One control participant had missing data, resulting in n = 51 for these comparisons. “Don’t know” responses are presented for transparency but were excluded from statistical testing. No significant differences were observed between groups. Con – controls, Mig – migraineurs, n – number of individuals, U - Mann-Whitney U test statistic, χ² – Chi-squared statistic.

**Table 2. Population descriptors of transcriptomic cohort - history of allergy**

|  |  | n= Con(Mig) | Pollen, weed, dust | Drug | Food | X^2^ | p-value |
| --- | --- | --- | --- | --- | --- | --- | --- |
| Allergy | Male | 4(2) | 4(7) | 3(2) | 1(0) | 0.61 | 0.44 |
|  | Female | 3(7) |  |  |  |  |  |

No significant difference could be demonstrated between groups as evidenced by chi-square tests. n – number of individuals, Con – controls, Mig – migraineurs.

**Table 3. Population descriptors of transcriptomic cohort - smoking status**

|  |  | n= Con(Mig) | 1 | 2 | 3 | 4 | 5 | U | p-value |
| --- | --- | --- | --- | --- | --- | --- | --- | --- | --- |
| Smokers | Male | 5(5) | 23(0) | 4(14) | 0(4) | 2(4) | 1(0) | 90 | 1.98E-06 |
|  | Female | 2(17) |  |  |  |  |  |  |  |

Significant differences could be detected based on the Mann-Whitney U-test. n – number of individuals, Con – controls, Mig – migraineurs, 1 - never, 2 - occasionally, 3- a few stems/day, 4- half box/day, 5- one or more than one box/day, U - Mann-Whitney U test statistic.

**Table 4. Population descriptors of transcriptomic cohort - supplement intake**

|  |  | n= Con(Mig) | Contra- ceptives | Vit | Trace elements | Anti- hist | O3 | Homeo- pathics | Others |
| --- | --- | --- | --- | --- | --- | --- | --- | --- | --- |
| Drugs | Male | 4(0) | 4(6) | 8(0) | 1(3) | 2(0) | 2(0) | 1(0) | 4(2) |
|  | Female | 10(9) |  |  |  |  |  |  |  |

All participants reported supplement intake, vitamins (Vit) contained multivitamin-, vitamin A, -C and -D preparations, trace elements contained magnesium, iron, folic acid and zinc. Antihistamines (Antihist) were cetirizin and desloratidin, omega-3 (O3) was supplemented by cod liver oil and Omega-3 capsules. Homeopathic medicines were not specified, while others contain antacids, glycosaminoglycan, myo-inositol, nasal spray, steroid cream and Q10. n – number of individuals, Con – controls, Mig – migraineurs.

**Table 5. Population descriptors of transcriptomic cohort - contraceptive use**

|  | COC | Vaginal ring |
| --- | --- | --- |
| Contraceptive type | 9 | 1 |

All contraceptives used are hormonal, just one participant used vaginal ring, the rest used COC during the study. COC – Combined oral contraceptive pill.

**Descriptive statistics of the additional cohorts used in the study**

In the following detailed descriptive statistics are presented about additional cohorts used in genetic analyses.

**Table 6. UK Biobank sample characteristics**

| UK Biobank sample characteristics | | | |
| --- | --- | --- | --- |
| **Age** | Mean | 58.1743 |  |
|  | SE | 0.0167 |  |
|  | Range | 39-72 |  |
| **Smoking** | N | **Allergy** | N |
| Cases | 102976 | Cases | 25321 |
| Controls | 66403 | Controls | 144469 |
| **Sex** | N | **Migraine** | N |
| Males | 101766 | Cases | 6139 |
| Females | 98163 | Controls | 193790 |
| **Retinol equivalent intake** | Mean | 978.9086 |  |
|  | SE | 3.8709 |  |
|  | Range(μg) | 0-41446 |  |
| **Retinol intake** | Mean | 482.1076 |  |
|  | SE | 3.4461 |  |
|  | Range(μg) | 0-38784 |  |

Sample characteristics of the UK Biobank cohort used in the genetic analyses of LEGs and vitamin A related SNP-level analyses. N – number of individuals, SE – standard error, μg – micrograms.

**Table 7. Migraine transcriptomic cohort sample characteristics**

| Migraine transcriptomic cohort sample characteristics | | | |
| --- | --- | --- | --- |
| **Age** | Mean | 27.5051 |  |
|  | SE | 0.3868 |  |
|  | Range | 19-49 |  |
| **Smoking** | Mean | 4.6329 |  |
|  | SE | 0.0511 |  |
|  | Range | 1-5 |  |
| **Sex** | N | **Migraine** | N |
| Males | 83 | Cases | 172 |
| Females | 206 | Controls | 117 |
| **Allergy** | N |  |  |
| Yes | 106 |  |  |
| No | 177 |  |  |

Sample characteristics of the migraine transcriptomic cohort used in the genetic analyses of LEGs and vitamin A related SNP-level analyses. N – number of individuals, SE – standard error

**Table 8. Results of the comparison of retinol and vitamin A retinol equivalent intake between migraine and control group**

|  | Retinol mean | t-test p-value | Vitamin A retinol equivalent mean | t-test p-value |
| --- | --- | --- | --- | --- |
| migraine | 426.7946 | 0.00021 | 946.5640 | 0.06491 |
| control | 484.0020 |  | 980.0163 |  |

**Results of allergy- and sex dependent analyses**

We considered two remaining phenotypic factors, allergy and female/male sex, that may have influenced our findings. On one hand, correction for allergy - despite its overall beneficial effect on replicability – may have removed migraine-relevant genes from comparisons due to the high comorbidity and potential shared aetiology with migraine [1]. We tested this possibility by comparing results of allergy-corrected and -uncorrected analyses. On the other hand, migraine prevalence is two- to threefold in females compared to males [2]. Correction for sex in our previous tests has ensured that the identified core results are important in both sexes. Nonetheless, sex-specific analyses are recommended in migraine research [2] and we speculated that analyses comparing the sexes may deliver additional insights about the stability of the found genes and pathways and can point to sex-specific characteristics for future studies.

Allergy-uncorrected analysis yielded one significantly downregulated, replicated gene, *CYP26B1* (FDR_S1_ = 0.038, log2FC_S1_ = -6.774, FDR_S2_ = 0.009, log2FC_S2_ = -6.698) and 298 significantly enriched, replicated Gene Ontology (GO) molecular function (MF) and GO biological process (BP) pathways (Online resource 7,52), with notable differences in cardiovascular- and hemostasis-related pathways and 6 positively enriched pathways related e.g. to nasal mucosa and rhinitis in comparison with allergy-corrected results. Only 6 pathways (from the 88) became non-significant or could not be replicated from the core findings: *cellular oxidant detoxification*, *defence response to bacterium*, *antioxidant activity*, *antimicrobial humoral immune response mediated by antimicrobial peptide*, *cellular response to toxic substance*, *regulation of protein targeting* (Online resource 43).

All in all, gene- and pathway-level findings supported common, potentially pleiotropic mechanisms in allergy and migraine, which became non-significant due to allergy-correction. Such genes and pathways may serve as the basis for future studies examining shared pathomechanisms. At the same time 82 pathways, 53 LEGs (including all LEG5) and the significantly replicated *CYP26B1* gene were found to be stable, allergy-independent factors in MO.

Among sex-focused analyses, allergy-corrected analysis of female versus male migraine patients controlling for non-migraine samples within sexes using the GO gene sets provided 33 positively, and 1 negatively enriched GOMF and GOBP sets. Thirteen among these significant, replicated pathways showed the same NES sign in sex-specific analyses and were significant or close to significance in the migraine versus control comparison too. The remaining 22 showed opposite directions of enrichment and lack of replicable (nominal) significance in females (Online resources 14,43,52,54). Accordingly, overlap between the FDR-significant pathway sets showed that migraine and control comparisons contained 85.00% of the 20 pathways from the female migraine versus female control, and 46.77% of the 62 pathways from the male migraine versus male control comparisons (Online resources 43,52), suggesting that significance in female migraine versus female controls acted as filter for the general comparison of migraine versus controls. Analysis of the LEGs of the female migraine versus male migraine comparison showed that *PRTN3*, *CCL23* and *CTSG* are LEGs behind the significantly different GOMF/GOBP pathways between female migraine versus male migraine with mean absolute log2FC larger than 2 between the sexes. *CYP26B1* and *CORIN* were notably missing, confirming their lack of contribution to pathway-level sex-differences. Analysis of the LEG5 comparing migraine with controls in the sexes separately, indicated substantial sex-dependence in the magnitude of changes (but not in the direction) of *PRTN3*, *CCL23* and *CTSG*, with *PRTN3* and *CTSG* being less differentially expressed in men and *CCL23* in women (Online resource 72). Other genes of LEG69 also showed sex-dependent variations (Online resource 38,72). *CYP26B1* and *CORIN* were the only genes with large downregulations of nominal significance (p<0.05) in both sexes at all time points, indicating that many among the gene-level expression changes have relevance for pathways both for migraine in general and for sex-specific migraine-related variation.

All in all sex-specific analyses confirmed that 1) migraine versus control comparison contains pathways relevant in both sexes, 2) there are (female- and) male-specific pathways worth to explore in future investigations, 3) pathway- and gene-level changes in the sexes often show same direction, but different magnitudes and 4) CYP26B1 and CORIN are sex-independent genes behind MO-relevant pathways (for a list of likely sex- and history of allergy independent pathways and LEGs see Online resource 38).

**Supplementary methods**

**Power Calculations**

In order to assess the power of the experiment to discover differentially expressed genes, we calculated power under the following assumptions: 1) sample size was fixed (nmigraine = 22, ncontrol = 30), 2) gene counts follow a negative binomial distribution, 3) alpha threshold, below which we consider significant differences was set to 0.05, 4) variables were, a) Log2FC, representing the log2 fold change difference between control and diseased groups varied with 0.5, 1, 2, 3, 4, 5, 6 values, b) dispersion parameter sigma varied between 0.4 and 0.8 with a step size of 0.08, in accordance with empirically determined trend-level dispersion estimates (see Figure 1) and c) mean expression count empirically determined from our dataset (with a median of the averages being 77.76 counts across all samples at both time points) varied from 5, 25 with step size 1, from 25 to 50 with step size 5, and from 50 to 110 with step size 10, and 5) we use a negative binomial regression like that in edgeR to (re)discover differentially expressed genes, assuming that dispersion estimates are accurately modeled (in our case identical with the one for the sampling). In short, we have built a model to test, if the distributional (statistical) properties of the data are valid, could we have enough power to detect significant differences.

In accordance with the above assumptions, we sampled a random variable from the negative binomial distribution using the following Poisson-Gamma mixed parametrization, where

$x\text{\textasciitilde}Poisson\left( \theta\right)\text{, with }\theta\text{ \textasciitilde}\Gamma\left( 1/\sigma,\mu*\sigma\right)$

This mixed parametrization results in a random variable with:

$$mean\left( x \right)=\mu$$

$$var\left( x \right)=\mu+\sigma*\mu^{2}$$

EdgeR assumes about counts for gene *g*:

$$var\left( g_{i} \right)=\mu_{g_{i}}+\sigma_{g}*\mu_{g_{i}}^{2}$$

, where *g_i_* is gene *i*, μ the mean expression count, σ is the dispersion parameter. Therefore, the sampling parameterized above and the negative binomial regression in edgeR have the same underlying statistical assumptions. We sampled the above NB2 distribution for both control and migraine patients such that migraine patient’s mean gene counts were log2FC times larger than the control group’s. Then, the negative binomial regression model was used to test how many times we can recover significant differences (using the threshold alpha for significance) from 1000 sampling for the given log2FC and sigma parameters for a single gene. The resulting proportion was the power for the given difference and dispersion parameters.

We show power with the mean counts given the different values of log2FC and dispersion (sigma, see Figure 9). (We have to note, that low-expression genes were filtered with the default settings of edgeR’s filterbyexpr function in our experiment).


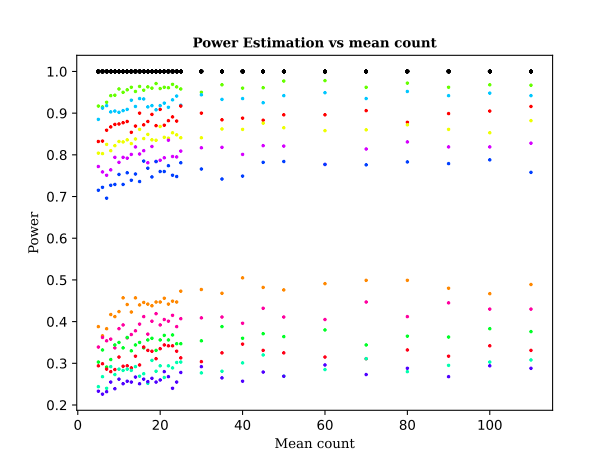

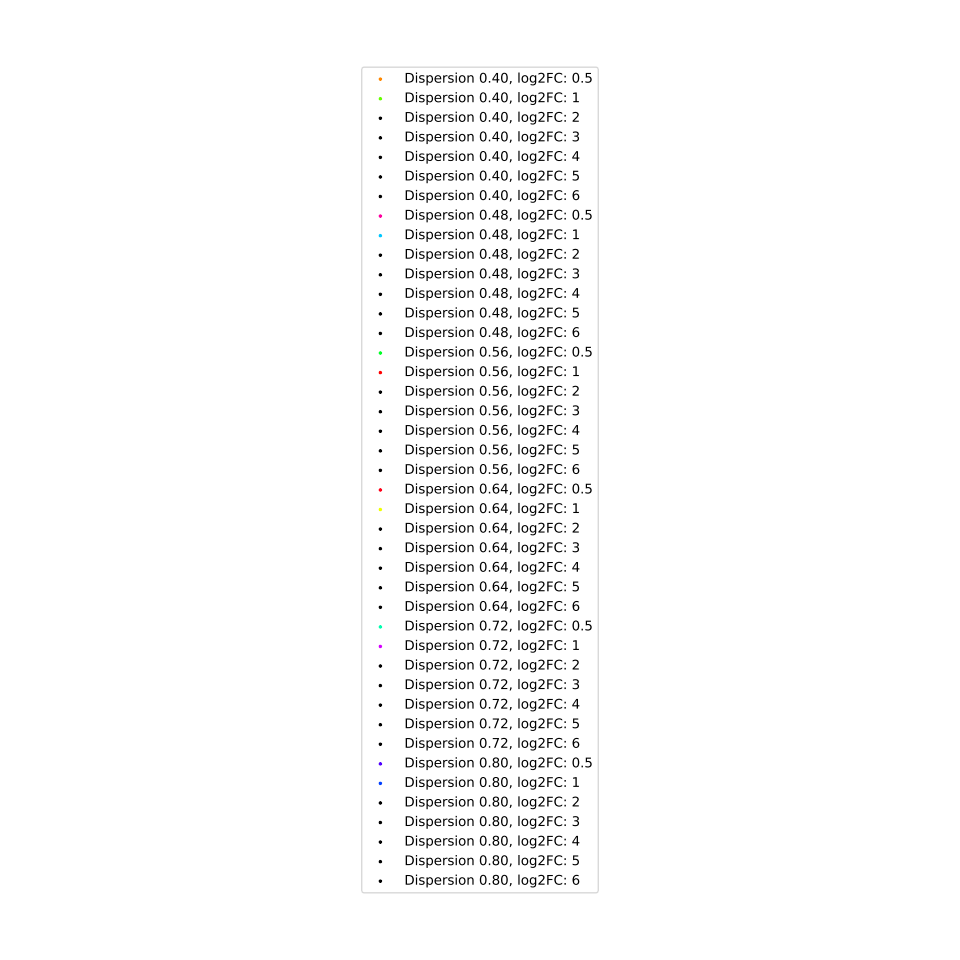


**Figure 9. Power estimate versus mean count plot**

We have modeled counts according to the negative binomial distribution for control and migraine samples and tried to recover differentially expressed genes using the negative binomial regression. Given the real sizes of patients and controls, in most cases we have close 100% power to detect relevant differences (black line at 1). The only case where our power decreases is when log2FC is small and dispersion is large, although even in this case we should be have been able to detect significant differences in about one third of the cases.

The above power estimates indicate that we were able to detect changes with sigma below 0.72 and log2FC larger than 1 with about 80% power, while for log2FC >= 2 we had almost 100% power to detect significant differences (see black lines).

The negative binomial distribution adequately models count data in gene expression experiments, thus, it is reasonable to assume that the lack of significant findings in the present work may be due to 1) other migraine-influencing factors, like physical activity, diet, etc. increasing variance, 2) changes being not gene-driven (rather e.g. pathway-driven with differences in individual genes being smaller than the log2FC threshold), 3) only genes with very low gene expression or very large variance playing a role in MO (for which we did not have enough power). Either way, the present study, given our strict selection criteria and rigorous co-variate corrections, was reasonably assumed to be well-powered for the detection of differences in a wide range of conditions.

We have to note, that the above calculations are valid for the gene-level significance tests for a single gene. For LEGs we conducted no extra tests, that was a post-hoc filtering of factors already important for significantly replicated pathways assessed by gene set enrichment analyses.

We also did not simulate large number of genes, albeit FDR correction is known to reduce power [3], because we did not assume that this correction would be the main reason for the lack of significant findings in our study. As a further note, the sample sizes in our work correspond to a very-well powered study according to [3] using similar statistical assumptions.

**AutoDock Vina and machine learning-based drug binding predictions**

In the following two schematic figures are presented about the methods used to assess drug binding to proteins corresponding to the genes.


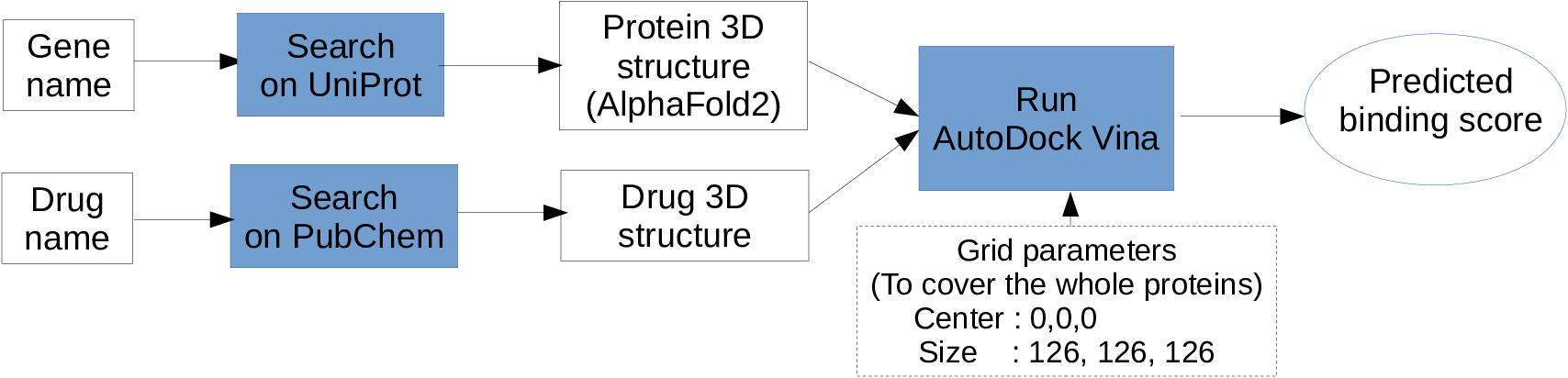


**Figure 10. Schematic workflow of AutoDock Vina binding calculations between drugs and genes**

The figure shows from left to right the flow from input to predicted binding scores. Input is a gene and a drug name, from which protein 3D structures and drug structures were obtained through UniProt’s AlphaFold 2 structure and PubChem, respectively. With these structures as input AutoDock Vina was run after the prepare_receptor -A and prepare_ligand commands with the described parameters. Predicted binding scores were evaluated and lowest scores (strongest binding) were considered as the binding score between the protein and the drug.


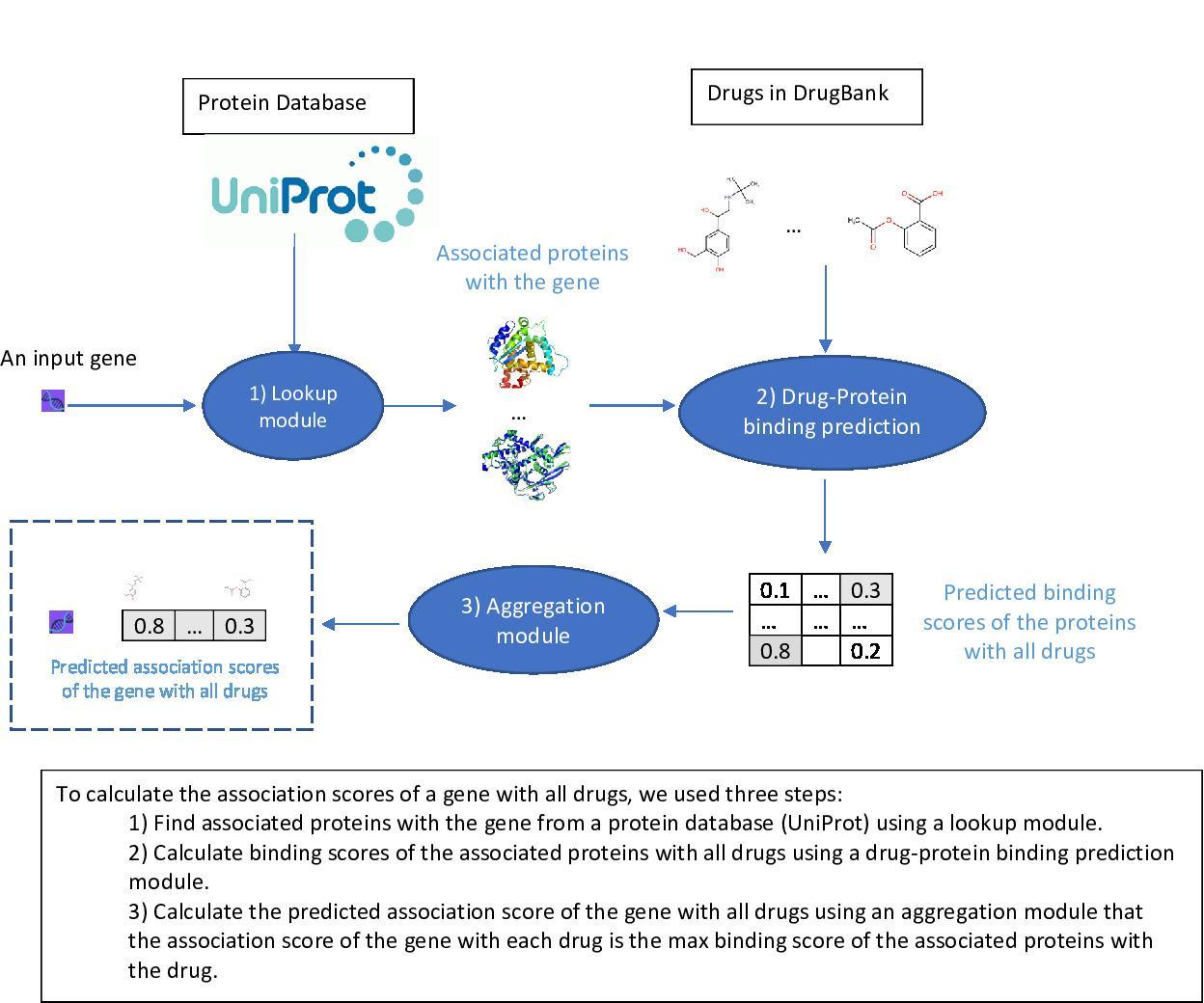


**Figure 11. Schematic workflow of machine learning based drug-protein binding predictions**

The figure shows the schematic workflow to obtain drug-protein binding scores in large-scale analysis of DrugBank drugs to gene expression-level alterations. For detailed steps see the 3 points at the bottom of the figure.

**Supplementary References**

[1] Duse DC, Reed ML, Fanning KM, Bostic R, Dodick DW, Schwedt TJ, Munjal S, Singh P, Lipton RB. Comorbid and co-occurring conditions in migraine and associated risk of increasing headache pain intensity and headache frequency: results of the migraine in America symptoms and treatment (MAST) study. J Headache Pain. 2020;21(1):23. https://doi.org/10.1186/s10194-020-1084-y

[2] Vetvik KG, MacGregor EA. Sex differences in the epidemiology, clinical features, and pathophysiology of migraine. Lancet Neurol. 2017;16(1):76–87. https://doi.org/10.1016/S1474-4422(16)30293-9

[3] Li X, Wu D, Cooper NGF, Rai SN. Sample size calculations for the differential expression analysis of RNA-seq data using a negative binomial regression model. Stat Appl Genet Mol Biol. 2019;18(1):20180021. https://doi:10.1515/sagmb-2018-0021.
